# Systems Biology Graphical Notation: Process Description language Level 1 Version 2.0

**DOI:** 10.1515/jib-2019-0022

**Published:** 2019-06-13

**Authors:** Adrien Rougny, Vasundra Touré, Stuart Moodie, Irina Balaur, Tobias Czauderna, Hanna Borlinghaus, Ugur Dogrusoz, Alexander Mazein, Andreas Dräger, Michael L. Blinov, Alice Villéger, Robin Haw, Emek Demir, Huaiyu Mi, Anatoly Sorokin, Falk Schreiber, Augustin Luna

**Affiliations:** Biotechnology Research Institute for Drug Discovery, AIST, Tokyo135-0064, Japan; Computational Bio Big-Data Open Innovation Laboratory (CBBD-OIL), AIST, Tokyo 169-8555, Japan; Department of Biology, Norwegian University of Science and Technology (NTNU), Trondheim, Norway; Eight Pillars Ltd, 19 Redford Walk, Edinburgh EH13 0AG, UK; European Institute for Systems Biology and Medicine, CIRI UMR5308, CNRS-ENS-UCBL-INSERM, Université de Lyon, 50 Avenue Tony Garnier, 69007 Lyon, France; Faculty of Information Technology, Monash University, Melbourne, Australia; Department of Computer and Information Science, University of Konstanz, Konstanz, Germany; Computer Engineering Department, Bilkent University, Ankara 06800, Turkey; i-Vis Research Lab, Bilkent University, Ankara 06800, Turkey; Luxembourg Centre for Systems Biomedicine, University of Luxembourg, 6 Avenue du Swing, L-4367 Belvaux, Luxembourg; Institute of Cell Biophysics, Russian Academy of Sciences, 3 Institutskaya Street, Pushchino, Moscow Region, 142290, Russia; Computational Systems Biology of Infection and Antimicrobial-Resistant Pathogens, Center for Bioinformatics Tübingen (ZBIT), 72076 Tübingen, Germany; Department of Computer Science, University of Tübingen, 72076 Tübingen, Germany; German Center for Infection Research (DZIF), partner site Tübingen, Tübingen, Germany; Center for Cell Analysis and Modeling, UConn Health, Farmington CT 06030, USA; Freelance IT Consultant, Brighton, UK; Ontario Institute for Cancer Research, MaRS Centre, Toronto, Ontario, Canada; Computational Biology Program, Oregon Health and Science University, Portland, Oregon, USA; Oregon Health and Science University, Department of Molecular and Medical Genetics, Portland, Oregon, USA; Department of Preventive Medicine, Keck School of Medicine of USC, University of Southern California, Los Angeles, CA 90033, USA; cBio Center, Department of Data Sciences, Dana-Farber Cancer Institute, Boston, MA 02215, USA; Department of Cell Biology, Harvard Medical School, Boston, MA 02115, USA

**Keywords:** biological network, circuit diagram, SBGN, standard, systems biology, visualisation

## Abstract

The Systems Biology Graphical Notation (SBGN) is an international community effort that aims to standardise the visualisation of pathways and networks for readers with diverse scientific backgrounds as well as to support an efficient and accurate exchange of biological knowledge between disparate research communities, industry, and other players in systems biology. SBGN comprises the three languages Entity Relationship, Activity Flow, and Process Description (PD) to cover biological and biochemical systems at distinct levels of detail. PD is closest to metabolic and regulatory pathways found in biological literature and textbooks. Its well-defined semantics offer a superior precision in expressing biological knowledge. PD represents mechanistic and temporal dependencies of biological interactions and transformations as a graph. Its different types of nodes include entity pools (e.g. metabolites, proteins, genes and complexes) and processes (e.g. reactions, associations and influences). The edges describe relationships between the nodes (e.g. consumption, production, stimulation and inhibition). This document details Level 1 Version 2.0 of the PD specification, including several improvements, in particular: 1) the addition of the equivalence operator, subunit, and annotation glyphs, 2) modification to the usage of submaps, and 3) updates to clarify the use of various glyphs (i.e. multimer, empty set, and state variable).

Automatically generated rough PDF by *ProofCheck* from River Valley Technologies Ltd

## Systems Biology Graphical Notation: Process Description language Level 1

### Version 2.0

May 1, 2019

**Editors**:

| | |
|---|---|
| Adrien Rougny | *AIST, Japan* |
| Vasundra Touré | *NTNU, Norway* |
| Stuart Moodie | *Eight Pillars Ltd, UK* |
| Irina Balaur | *EISBM, France* |
| Tobias Czauderna | *Monash University, Australia* |
| Hanna Borlinghaus | *University of Konstanz, Germany* |
| Ugur Dogrusoz | *Bilkent University, Turkey* |
| Alexander Mazein | *University of Luxembourg, Luxembourg* |
| Andreas Dräger | *University of Tübingen, Germany* |
| Michael Blinov | *UConn School of Medicine, USA* |
| Alice Villéger | *Freelance IT Consultant, UK* |
| Robin Haw | *Ontario Institute for Cancer Research, Canada* |
| Emek Demir | *OHSU, USA* |
| Huaiyu Mi | *University of Southern California, USA* |
| Anatoly Sorokin | *Institute of Cell Biophysics RAS, RU* |
| Falk Schreiber | *University of Konstanz, Germany* |
| Augustin Luna | *Dana-Faber Cancer Institute, USA* |

*To discuss any aspect of SBGN, please send your messages to the mailing list* sbgn-discuss@googlegroups.com*. To get subscribed to the mailing list or to contact us directly, please write to* sbgn-editors@googlegroups.com*. Bug reports and specific comments about the specification should be entered in the issue tracker* https://github.com/sbgn/process-descriptions/issues.

SBGN

## Preface

### Acknowledgements

The authors are grateful to all the attendees of the SBGN meetings, as well as to the subscribers of the sbgn-discuss@googlegroups.com mailing list. The authors would like to acknowledge especially the help of Frank T. Bergmann, Sarala Dissanayake, Ralph Gauges, Peter Ghazal, and Lu Li. Stuart Moodie and Anatoly Sorokin would like to acknowledge Igor Goryanin whose financial support and encouragement enabled us to commit the necessary time to the development of SBGN. Augustin Luna would also like to acknowledge Chris Sander for his financial support towards the development of this specification. A more comprehensive list of people involved in SBGN development is available in appendix 5 along with acknowledgement of financial support.

## Contents

**Preface** ii

**1 Introduction** 1
1.1 What are the languages? 1
1.2 Nomenclature 1
1.3 SBGN levels and versions 2
1.4 Developments, discussions, and notifications of updates 2
1.5 Note on the typographical convention 2

**2 Process Description Glyphs** 3
2.1 Overview 3
2.2 Controlled vocabularies used in SBGN Process Description Level 1 5
2.2.1 Entity pool node material types 6
2.2.2 Entity pool node conceptual types 6
2.2.3 Macromolecule covalent modifications 6
2.2.4 Physical characteristics 7
2.2.5 Cardinality 7
2.3 Auxiliary units 7
2.3.1 Glyph: *Unit of information* 8
2.3.2 Glyph: *State variable* 9
2.3.3 Glyphs: *Clone markers* 10
2.3.4 Glyphs: *Subunits* 12
2.3.5 Glyph: *Submap terminal* 14
2.4 Entity pool nodes 15
2.4.1 Glyph: *Unspecified entity* 15
2.4.2 Glyph: *Simple chemical* 16
2.4.3 Glyph: *Macromolecule* 16
2.4.4 Glyph: *Nucleic acid feature* 17
2.4.5 Glyph: *Multimer* 18
2.4.6 Glyph: *Complex* 20
2.4.7 Glyph: *Empty Set* 21
2.4.8 Glyph: *Perturbing agent* 21
2.4.9 Examples of complex EPNs 22
2.5 Defined sets of entity pool nodes 23
2.5.1 Glyph: *Compartment* 23
2.6 Process nodes 24
2.6.1 Glyph: *Process* 25
2.6.2 Glyph: *Omitted process* 27
2.6.3 Glyph: *Uncertain process* 28
2.6.4 Glyph: *Association* 29
2.6.5 Glyph: *Dissociation* 30
2.6.6 Glyph: *Phenotype* 31
2.7 Flux arcs 32
2.7.1 Glyph: *Consumption* 32
2.7.2 Glyph: *Production* 33
2.8 Modulation arcs 34
2.8.1 Glyph: *Modulation* 34
2.8.2 Glyph: *Stimulation* 35
2.8.3 Glyph: *Catalysis* 35
2.8.4 Glyph: *Inhibition* 36
2.8.5 Glyph: *Necessary stimulation* 36
2.9 Logical operators 38
2.9.1 Glyph: *And* 38
2.9.2 Glyph: *Or* 39
2.9.3 Glyph: *Not* 39
2.9.4 Glyph: *Equivalence* 40
2.10 Logic arc 41
2.10.1 Glyph: *Logic arc* 41
2.11 Annotating nodes and arcs 42
2.11.1 Glyph: *Annotation* 42
2.12 Referring to other nodes 43
2.12.1 Glyph: *Tag* 43
2.12.2 Glyph: *Equivalence arc* 44
2.13 Encapsulation 44
2.13.1 *Submap* 44

**3 Process Description Language Grammar** 47
3.1 Overview 47
3.2 Concepts 47
3.3 The conceptual model 48
3.4 Syntax 50
3.4.1 Node connectivity 50
3.4.2 Containment definition 51
3.5 Semantic rules 51
3.5.1 EPNs 51
3.5.2 Process Nodes 52
3.5.3 Cloning 54
3.5.4 Compartment spanning 54
3.5.5 Submaps 54
3.5.6 Equivalence operator 55

**4 Layout Rules for a Process Description** 56
4.1 Introduction 56
4.2 Requirements 56
4.2.1 Node-node overlaps 56
4.2.2 Node-edge crossing 57
4.2.3 Node border-edge overlaps 57
4.2.4 Edge-edge overlaps 57
4.2.5 Node orientation 57
4.2.6 Node-edge connection 57
4.2.7 Node labels 58

4.2.8 Edge labels . . . . . . . . . . 58
4.2.9 Compartments . . . . . . . . 58
4.3 Recommendations . . . . . . . . . . . 59
4.3.1 Node-edge crossing . . . . . 59
4.3.2 Labels . . . . . . . . . . . . . 59
4.3.3 Avoid edge crossings . . . . 59
4.3.4 Branching of *association* and *dissociation* . . . . . . . 59
4.3.5 Units of information . . . . 59
4.4 Additional suggestions . . . . . . . 59

**5 Acknowledgements 61**
5.1 Level 1 Release 1.0 . . . . . . . . . 61
5.2 Level 1 Release 1.1 . . . . . . . . . 61
5.3 Level 1 Release 1.2 . . . . . . . . . 61
5.4 Level 1 Release 1.3 . . . . . . . . . 61
5.5 Level 1 Release 2.0 . . . . . . . . . 61
5.6 Comprehensive list of acknowledgements . . . . . . . . . . . . . . . . . . 62
5.7 Financial Support . . . . . . . . . . 62

**A Complete examples of Process Description Maps 63**

**B Examples of use of the *equivalence operator* 68**

**C Reference card 74**

**D Issues postponed to future levels 76**
D.1 Multicompartment entities . . . . . 76
D.2 Logical combination of state variable values . . . . . . . . . . . . . . 76
D.3 Non-chemical entity nodes . . . . . 76
D.4 State and transformation of compartments . . . . . . . . . . . . . . 77

**E Revision History 78**
E.1 Version 1.0 to Version 1.1 . . . . . . 78
E.2 Version 1.1 to Version 1.2 . . . . . . 79
E.3 Version 1.2 to Version 1.3 . . . . . . 79
E.4 Version 1.3 to Version 2.0 . . . . . . 79

## Chapter 1

## Introduction

With the rise of systems and synthetic biology, the use of graphical representations of pathways and networks to describe biological systems has become pervasive. It was, therefore, inherent to use a consistent notation that would allow people to interpret those maps easily and quickly, without the need for extensive legends. Furthermore, distributed investigation of biological systems in different labs as well as activities such as synthetic biology, which reconstruct biological systems, need to exchange their descriptions unambiguously, as engineers exchange circuit diagrams. The goal of the Systems Biology Graphical Notation (SBGN) is to standardise the graphical/visual representation of biochemical and cellular processes. SBGN defines comprehensive sets of symbols with precise semantics, together with detailed syntactic rules defining their use. It also describes the manner in which such graphical information should be interpreted. SBGN is made up of three different and complementary languages [1]. This document defines the *Process Description* visual language of SBGN. Process Descriptions are one of three views of a biological process offered by SBGN. It is the product of many hours of discussion and development by many individuals and groups.

### 1.1 What are the languages?

The **Process Description** language permits the description of all the processes taking place in a biological system. The **Entity Relationship** language permits the description of all the relations involving the entities of a biological system. The **Activity Flow** language permits the description of the flow of activity in a biological system.

### 1.2 Nomenclature

The three languages of SBGN should be referred to as:

- the Process Description language (the PD language).
- the Entity Relationship language (the ER language).
- the Activity Flow language (the AF language).

A specific representation of a biological system in one of the SBGN languages should be referred to as:

- a Process Description map (a PD map).
- an Entity Relationship map (an ER map).
- an Activity Flow map (an AF map).

The corpus of all SBGN representations should be referred to as:

- Process Descriptions.
- Entity Relationships.
- Activity Flows.

The capitalisation is important. PD, ER and AF are names of languages. As such they must be capitalised in English. This is not the case of the accompanying noun (language or map).

### 1.3 SBGN levels and versions

It was unquestionable at the outset of SBGN development that it would be impossible to design a perfect and complete notation right from the beginning. Apart from the prescience, this would require (which, sadly, none of the authors possesses), it also would likely need a broad language that most newcomers would shun as being too involved. Thus, the SBGN community followed an idea used in the development of other standards, i.e., stratify language development into levels.

A *level* of one of the SBGN languages represents a set of features deemed to fit together cohesively, constituting a useful set of functionality that the user community agrees sufficient for a reasonable set of tasks and goals. Within *levels*, *versions* represent a small evolution of a language, that may involve new glyphs, clarified semantics, but no fundamental change of the way maps are to be generated and interpreted. Moreover, new versions should be backwards compatible, i.e., Process Description maps that conform to an earlier version of the Process Description language within the same level should still be valid. This does not apply to a new level. Capabilities and features that cannot be agreed upon and are judged insufficiently critical to require inclusion in a given level are postponed to a higher level or version. In this way, the development of SBGN languages is envisioned to proceed in stages, with each higher levels adding richness compared to the levels below it.

### 1.4 Developments, discussions, and notifications of updates

The SBGN website (http://sbgn.org/) is a portal for all things related to SBGN. It provides a web forum interface to the SBGN discussion list (sbgn-discuss@googlegroups.com) and information about how anyone may subscribe to it. The easiest and best way to get involved in SBGN discussions is to join the mailing list and participate.

Face-to-face meetings of the SBGN community are announced on the website as well as the mailing list. Although no set schedule currently exists for workshops and other meetings, we envision holding at least one public workshop per year. As with other similar efforts, the workshops are likely to be held as satellite workshops of larger conferences, enabling attendees to use their international travel time and money more efficiently.

Notifications of updates to the SBGN specification are also broadcast on the mailing list and announced on the SBGN website.

### 1.5 Note on the typographical convention

The concept represented by a glyph is written using a regular font, while a *glyph* means the SBGN visual representation of the concept. For instance "a biological process is encoded by the SBGN PD *process*."

## Chapter 2

## Process Description Glyphs

### 2.1 Overview

To set the stage for what follows in this chapter, we give first a brief overview of some of the concepts in the Process Description language with the help of an example shown in Figure 2.1 on the following page.

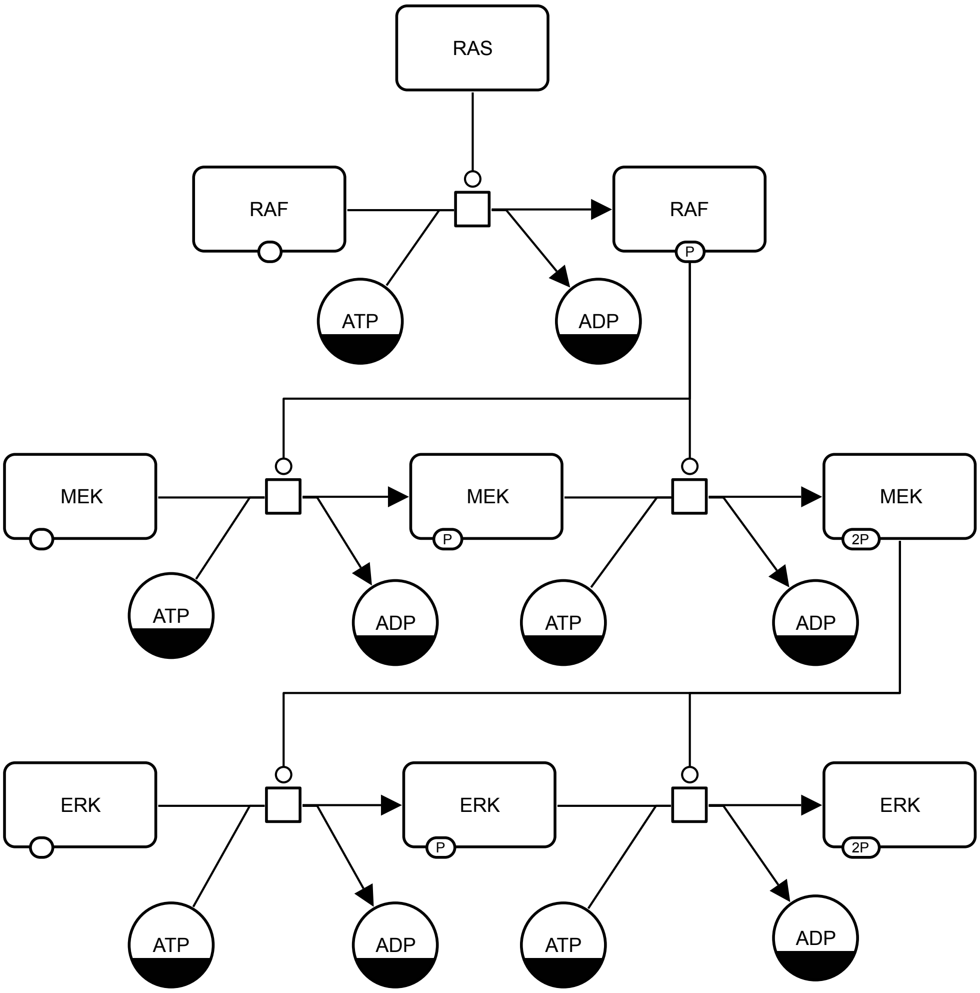

**Figure 2.1:** *This example of a Process Description map uses two kinds of entity pool nodes: one for pools of different macromolecules (Section 2.4.3) and another for pools of simple chemicals (Section 2.4.2). Most* macromolecule *nodes in this map are adorned with* state variables *(Section 2.3.2) representing phosphorylation states. This map uses one type of* process node, *the* process *node (Section 2.6.1), and three kinds of connecting arc,* consumption *(Section 2.7.1),* production *(Section 2.7.2) and* catalysis *(Section 2.8.3). Finally, some entity pool nodes have dark bands along their bottoms; these are* clone markers *(Section 2.3.3) indicating that the same pool nodes appear multiple times in the map.*

The map in Figure 2.1 is a simple map for part of a mitogen-activated protein kinase (MAPK) cascade. The larger nodes in the figure (some of which are in the shape of rounded rectangles and others in the shape of circles) represent biological materials—things like macromolecules and simple chemicals. The biological materials are altered via processes, which are indicated in Process Description language by lines with arrows and other decorations. In this particular map, all of the processes happen to be the same: processes catalysed by biochemical entities. The directions of the arrows indicate the direction of the processes; for example, unphosphorylated RAF kinase processes to phosphorylated RAF kinase via a process catalysed by RAS. Although ATP and ADP are shown as incidental to the phosphorylations on this particular graph, they are involved in the same process as the proteins getting phosphorylated. The small circles on the nodes for RAF and other entity pools represent state variables (in this case, phosphorylation sites).

The essence of the Process Descriptions is the *change*: it shows how different entities in the system process from one form to another. The entities themselves can be many different things. In the example of Figure 2.1, they are pools of either macromolecules or simple chemicals, but as will become clear later in this chapter, they can be other conceptual and material constructs as well. Note also that we speak of *entity pools* rather than individuals; this is because, in biochemical network models, one does not focus on single molecules, but rather collections of molecules of the same kind. The molecules in a given pool are considered indistinguishable from each other. The way in which one type of entity is transformed into another is conveyed by a *process node* and links between entity pool nodes, and process nodes indicate influence by the entities on the processes. In the case of Figure 2.1 on the previous page, those links describe consumption Section 2.7.1, production Section 2.7.2 and catalysis Section 2.8.3, but others are possible. Finally, nodes in Process Descriptions are usually not repeated; if they do need to be repeated, they are marked with *clone markers*—specific modifications to the appearance of the node (Section 2.3.3). The details of this and other aspects of Process Description notation are explained in the rest of this chapter.

Table 2.1 summarizes the different SBGN abstractions described in this chapter.

| **Component** | **Abbrev.** | **Role** | **Examples** |
|---|---|---|---|
| Entity pool node | EPN | A population of entities that cannot be distinguished from each other | Specific macromolecules or other chemical species |
| Container node | CN | An encapsulation of one or more other SBGN constructs | Compartments |
| Process node | PN | A process that transforms one or more EPNs into one or more other EPNs | Process, association, dissociation |
| Arc | — | Links between EPNs, CNs or Logical Operators to PNs or Logical operators | Production, catalysis, inhibition |
| Logical operators | LO | Combines one or several inputs into one output | Boolean *and*, *or*, *not* |

**Table 2.1:** *Summary of Process Description components and their roles.*

### 2.2 Controlled vocabularies used in SBGN Process Description Level 1

Some glyphs in SBGN Process Descriptions can contain particular kinds of textual annotations conveying information relevant to the purpose of the glyph. These annotations are *units of information* (Section 2.3.1) or *state variables* (Section 2.3.2). For example, multimers can have a unit of information conveying the number of monomers composing the multimer. Other cases are described throughout the rest of this chapter.

The text that appears as the unit of information decorating an Entity Pool Node (EPN) must in most cases be prefixed with a controlled vocabulary term indicating the type of information being expressed. The prefixes are mandatory except in the case of macromolecule covalent modifications (Section 2.2.3). Without the use of controlled vocabulary prefixes, it would be necessary to have different glyphs to indicate different classes of information; this would lead to an explosion in the number of symbols needed.

In the rest of this section, we describe the controlled vocabularies (CVs) used in SBGN Process Description Level 1. They cover the following categories of information: an EPN's material type, an EPN's conceptual type, covalent modifications on macromolecules, the physical characteristics of compartments, and cardinality (e.g., of multimers). In each case, some CV terms are predefined by SBGN, but unless otherwise noted, *they are not the only terms permitted.* Users may use other CV values not listed here. In such cases, they should explain the term's meanings in a figure legend or other text accompanying the map. Users of CV values not listed here should *strongly* attempt different prefixes from those listed in this document.

#### 2.2.1 Entity pool node material types

The material type of an EPN indicates its chemical structure and physical composition. A list of common material types is shown in Table 2.2, but others are possible. The values are to be taken from the Systems Biology Ontology (`http://www.ebi.ac.uk/sbo/`), specifically from the branch having identifier `SBO:0000240` (the *material entity* under *entity*).

| Name | Label | SBO term |
|---|---|---|
| Non-macromolecular ion | `mt:ion` | `SBO:0000327` |
| Non-macromolecular radical | `mt:rad` | `SBO:0000328` |
| Ribonucleic acid | `mt:rna` | `SBO:0000250` |
| Deoxyribonucleic acid | `mt:dna` | `SBO:0000251` |
| Protein | `mt:prot` | `SBO:0000297` |
| Polysaccharide | `mt:psac` | `SBO:0000249` |

**Table 2.2:** *A sample of values from the* material types *controlled vocabulary (Section 2.2.1).*

The material types are in contrast to the *conceptual types* (see below). The distinction is that material types are about physical composition, while conceptual types are about roles. For example, a strand of RNA is a material type, but its use as messenger RNA is a role.

#### 2.2.2 Entity pool node conceptual types

An EPN's *conceptual type* indicates its function within the context of a given Process Description. A list of common conceptual types is shown in Table 2.3, but others are possible. The values are to be taken from the Systems Biology Ontology (`http://www.ebi.ac.uk/sbo/`), specifically from the branch having identifier `SBO:0000241` (the *conceptual entity* under *entity*).

| Name | Label | SBO term |
|---|---|---|
| Gene | `ct:gene` | `SBO:0000243` |
| Transcription start site | `ct:tss` | `SBO:0000329` |
| Gene coding region | `ct:coding` | `SBO:0000335` |
| Gene regulatory region | `ct:grr` | `SBO:0000369` |
| Messenger RNA | `ct:mRNA` | `SBO:0000278` |

**Table 2.3:** *A sample of values from the* conceptual types *vocabulary (Section 2.2.2).*

#### 2.2.3 Macromolecule covalent modifications

A common reason for the introduction of state variables (Section 2.3.2) on an entity is to allow access to the configuration of possible covalent modification sites on that entity. For instance, a macromolecule may have one or more sites where a phosphate group may be attached; this change in the site's configuration (i.e., being either phosphorylated or not) may factor into whether, and how, the entity can participate in different processes. Being able to describe such modifications consistently is the motivation for the existence of SBGN's covalent modifications controlled vocabulary.

Table 2.4 lists selected common types of covalent modifications. The most common values are defined by the Systems Biology Ontology in the branch having identifier `SBO:0000210` (*addition of a chemical group* under *interaction→process→biochemical or transport reaction→biochemical reaction→conversion*). The labels shown in Table 2.4 on the next page are defined by SBGN

Process Description Level 1; for all other kinds of modifications not listed here, the author of a Process Description must create a new label (and should also describe the meaning of the label in a legend or text accompanying the map).

| Name | Label | SBO term |
|---|---|---|
| Acetylation | Ac | SBO:0000215 |
| Glycosylation | G | SBO:0000217 |
| Hydroxylation | OH | SBO:0000233 |
| Methylation | Me | SBO:0000214 |
| Myristoylation | My | SBO:0000219 |
| Palmytoylation | Pa | SBO:0000218 |
| Phosphorylation | P | SBO:0000216 |
| Prenylation | Pr | SBO:0000221 |
| Protonation | H | SBO:0000212 |
| Sulfation | S | SBO:0000220 |
| Ubiquitination | Ub | SBO:0000224 |

**Table 2.4:** *A sample of values from the* covalent modifications *vocabulary (Section 2.2.3).*

#### 2.2.4 Physical characteristics

SBGN Process Description Level 1 defines a specific unit of information for describing particular common physical characteristics. Table 2.5 lists the particular values defined by SBGN Process Description Level 1. It is anticipated that these will be used to describe the nature of a *perturbing agent* (section 2.4.8) or a *phenotype* (section 2.6.6).

| Name | Label | SBO term |
|---|---|---|
| Temperature | pc:T | SBO:0000147 |
| Voltage | pc:V | SBO:0000259 |
| pH | pc:pH | SBO:0000304 |

**Table 2.5:** *A sample of values from the* physical characteristics *vocabulary (Section 2.2.4).*

#### 2.2.5 Cardinality

SBGN Process Description Level 1 defines a specific unit of information usable on multimers for describing the number of monomers composing the multimer. Table 2.6 on the next page shows the way in which the values must be written. Note that the value is a positive non-zero integer, and not (for example) a range. There is no provision in SBGN Process Description Level 1 for specifying a range in this context because it leads to problems of entity identifiability.

### 2.3 Auxiliary units

Auxiliary units are glyphs that decorate other glyphs, providing additional information that may be useful to the reader. In doing so, they change the meaning of the glyph or provide additional information about it. These can provide specific annotation (*unit of information*), state information (*state variable*), indicate duplication of entity pool nodes (*clone marker*), describe specific glyphs (*subunit* for *complex*), or provide handles to elements lying outside of the maps (*submap terminal* for *submap*).

| Name | Label | SBO term |
|---|---|---|
| cardinality | `N:#` | `SBO:0000364` |

**Table 2.6:** *The format of the possible values for the* cardinality *unit of information (Section 2.2.5). Here, # stands for the number; for example, "*`N:5`*".*

#### 2.3.1 Glyph: *Unit of information*

When representing biological entities, it is often necessary to convey some abstract information about the entity's function that cannot (or does not need to) be easily related to its structure. The *unit of information* is a decoration that can be used in this situation to add information to a glyph. Some example uses include: characterising a logical part of an entity such as a functional domain (a binding domain, a catalytic site, a promoter, etc.), or the information encoded in the entity (an exon, an open reading frame, etc.). A *unit of information* can also convey information about the physical environment, or the specific type of biological entity it is decorating.

**SBO Term:**
Not applicable.

**Incoming arcs:**
None.

**Outgoing arcs:**
None.

**Container:**
A *unit of information* is represented by a rectangular shape, as shown in Figure 2.2 on the following page. The centre of the shape should be placed on the border of the *EPN*.

**Label:**
A *unit of information* is identified by a label that is a string of characters that may be distributed on several lines to improve readability. The centre of the label must be placed on the centre of the container. The label may extend outside of the container. For certain predefined types of information having controlled vocabularies associated with them, SBGN defines specific prefixes that must be included in the text of the label and associated with the information's value to indicate the type of information in question. Together, a prefix and a value constitute the label. The controlled vocabularies predefined in SBGN Process Description Level 1 are described in Section 2.2 and summarised in the following list:

- `pc` container physical characteristic
- `mt` entity pool material type
- `ct` entity pool conceptual type
- `N` multimer cardinality

**Auxiliary items:**
None.

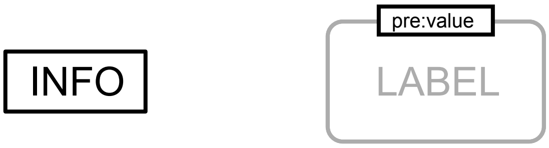

**Figure 2.2:** *The Process Description glyph for* unit of information*, shown plain on the left, and decorating a* macromolecule *(Section 2.4.3) on the right.*

#### 2.3.2 Glyph: *State variable*

Many biological entities such as molecules can exist in different states, meaning different physical or informational configurations. These states can arise for a variety of reasons. For example, macromolecules can be subject to post-synthesis modifications, wherein residues of the macromolecules (amino acids, nucleosides, or glucid residues) are modified through covalent linkage to other chemicals. Other examples of states are alternative conformations as in the closed/open/desensitised conformations of a transmembrane channel, and the active/inactive forms of an enzyme.

To describe such states, the Process Description introduces the concept of the state variable. A state variable of a biological entity usually has a name (e.g., "S122" to indicate residue Serine 122 of a protein), and can be assigned a value (e.g., "P", to indicate a phosphate group). Such a state variable models a dimension along which the state of the overall entity can vary. The state of an entity can then be described by the current values assigned to all its state variables, and of all its possible components, recursively. A state variable may be assigned no value; an example of a situation where this might arise is an unphosphorylated phosphorylation site. A state variable might also be unnamed, in cases where there is no ambiguity between this state variable and another state variable carried by the same entity (e.g., when an entity carries a unique state variable, it might be unnamed). In Process Description, state variables, together with the values assigned to them, are represented using the *state variable* glyph.

**SBO Term:**

Not applicable.

**Incoming arcs:**

None.

**Outgoing arcs:**

None.

**Container:**

A *state variable* is represented by a "stadium" shape, that is two semicircles of the same radius joined by parallel segments, as shown in Figure 2.3. The centre of the shape should be placed on the border of the EPN. In previous versions of this specification, the *state variable* was represented by an elliptic shape. This symbol is now **deprecated** in favour of the stadium shape described above.

**Label:**

A *state variable* is identified by a label that is a string of characters. The characters cannot be distributed on several lines. The centre of the label must be placed on the centre of the container. The label may extend outside of the container. The label is constituted of two substrings separated by the character "@", the first one indentifying the value of the state variable, and the second one its name. The character "@" is omitted when the state variable is unnamed. Aternatively, the substring identifying the name of the state variable may be displayed using a second label, placed outside of the shape. This is, however, strongly discouraged.

**Auxiliary items:**
None.

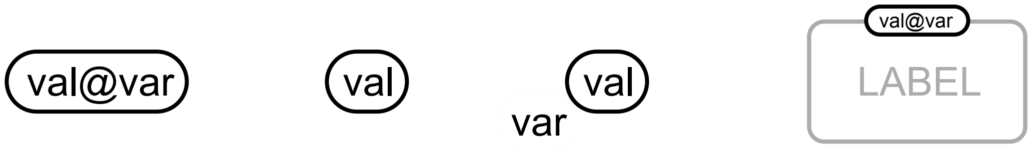

**Figure 2.3:** *The Process Description glyph for* state variable*, shown with a value and a variable on the far left, with only a value on the middle-left, with an additional label for the variable on the middle-right (discouraged), and decorating a* macromolecule *(Section 2.4.3) on the far right.*

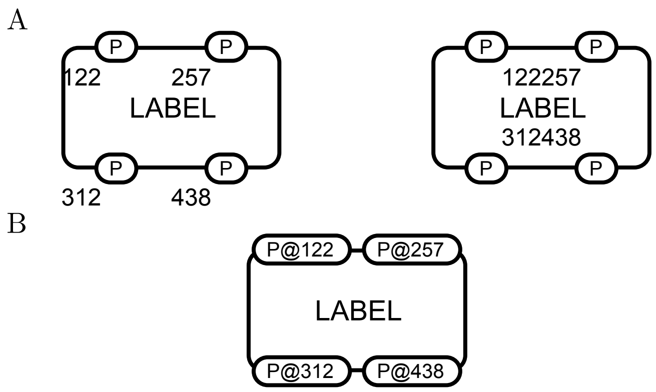

**Figure 2.4:** *A. Examples of discouraged use of* state variables*. B. Encouraged use.*

#### 2.3.3 Glyphs: *Clone markers*

If an *EPN* is duplicated on a map, it is necessary to indicate this fact by using the *clone marker* auxiliary unit. The purpose of this marker is to provide the reader with a visual indication that this node has been cloned, and that at least one other occurrence of the *EPN* can be found in the map (or in a submap; see Section 2.13.1). The clone marker takes two forms, simple and labelled, depending on whether the node being cloned can carry state variables (i.e., whether it is a stateful EPN). Note that an *EPN* belongs to a single compartment. If two glyphs labelled "X" are located in two different compartments, such as an EPN labelled "ATP" in the cytosol, and another EPN labelled "ATP" in the mitochondrial lumen, they represent different *EPNs* and therefore do not need to be marked as cloned.

##### *2.3.3.1* Simple clone marker

**SBO Term:**
Not applicable.

**Incoming arcs:**
None.

**Outgoing arcs:**
None.

**Container:**
A *simple clone marker* is represented by a portion of the surface of an *EPN* that has been modified visually through the use of a different shade, texture, or colour, as shown in Figure 2.5. The *simple clone marker* occupies the lower part of the *EPN*. The filled area must be smaller than the unfilled one.

**Label:**
None.

**Auxiliary items:**
None.

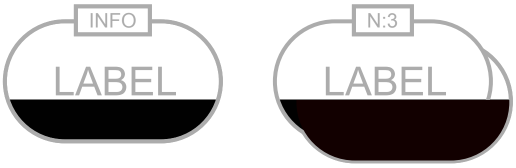

**Figure 2.5:** *The Process Description glyph for* simple clone marker *applied to a* simple chemical*and a* multimer *of* simple chemicals*.*

Figure 2.6 contains an example in which we illustrate the use of *simple clone markers* to clone the species ATP and ADP participating in different processes. This example also demonstrates the chief drawbacks of using clones: it leads to a kind of dissociation of the overall network and multiplies the number of nodes required, requiring more work on the part of the reader to interpret the result. Sometimes these disadvantages are offset in larger maps by a reduction in the overall number of line crossings, but not always. In general, we advise that cloning should be used sparingly.

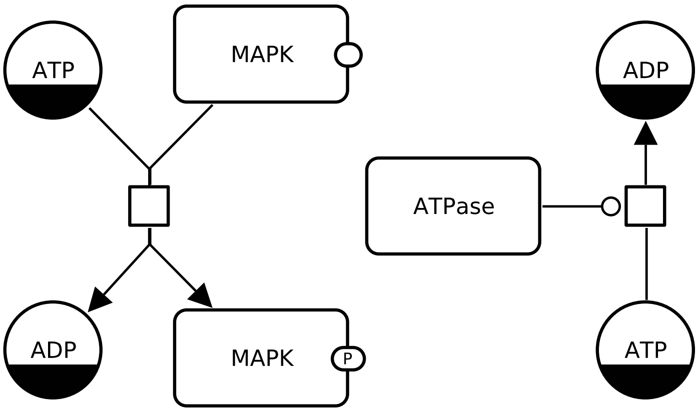

**Figure 2.6:** *An example of using cloning, here for the species ATP and ADP.*

##### 2.3.3.2 Labelled clone marker

Unlike the *simple clone marker*, the *labelled clone marker* includes (unsurprisingly, given its name) an identifying label that can be used to identify equivalent clones elsewhere in the map. This is particularly useful for stateful *EPNs* because these can have a large number of state variables displayed and therefore may be difficult to identify as being identical visually. All duplicated stateful EPNs must be decorated with a *labelled clone marker*.

**SBO Term:**
Not applicable.

**Incoming arcs:**
None.

**Outgoing arcs:**
None.

**Container:**
The *labelled clone marker* is represented by a portion of the surface of an *EPN* that has been modified visually through the use of a different shade, texture, or colour, as shown in Figure 2.7. The *labelled clone marker* occupies the lower part of the *EPN*. The filled area must be smaller than the unfilled one, but be large enough to accommodate the *labelled clone marker*'s label.

**Label:**
A *labelled clone marker* is identified by a label that is a string of characters that may be distributed on several lines to improve readability. The centre of the label must be placed on the centre of the container. The label may extend outside of the container. The font colour of the label and the colour of the *labelled clone marker* should contrast with one another. The label on a *labelled clone marker* is mandatory.

**Auxiliary items:**
None.

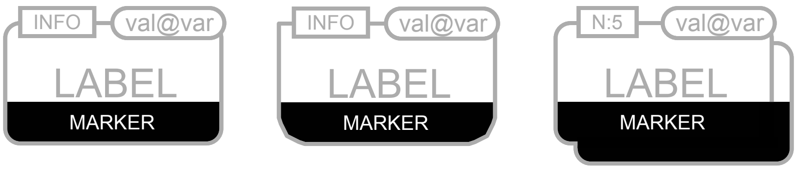

**Figure 2.7:** *The Process Description glyph for* labelled clone marker *applied to a* macromolecule*, a* nucleic acid feature *and a* multimer *of* macromolecules*.*

#### 2.3.4 Glyphs: *Subunits*

A complex is formed by the non-covalent binding of two or more entities, that become the subunits of the complex. In Process Description, the composition of a complex may be described using *subunit* glyphs, that are auxiliary units decorating *complexes*. *Subunits* do not represent or mimic entity pools (Section 2.4) and may only be used to represent the subunits included in a complex. The example in Figure 2.8 on page 14 illustrates the use of *subunits* to describe the composition of a complex. It also shows how the same complex can be represented without decorating *subunits*.

The SBGN Process Description defines nine different *subunit* glyphs, each representing a different type of bio-molecular (sub)-entity. The five main *subunits* are the *unspecified entity subunit*, *macromolecule subunit*, *simple chemical subunit*, *nucleic acid feature subunit*, and *complex subunit*. This latter *subunit* allows representing complexes formed of other complexes. The remaining four *subunits* are multimeric: *multimer of macromolecules subunit*, *multimer of simple chemicals subunit*, *multimer of nucleic acid feature subunit*, and *multimer of complexes subunit*.

**SBO Term:**
Not applicable.

**Incoming arcs:**
None.

**Outgoing arcs:**

None.

**Container:**

Each *subunit* is represented by its own shape depending on its bio-molecular nature, as shown in Table 2.7. Those shapes are the same as those used to represent entity pools (Section 2.4).

**Label:**

A *subunit* is identified by a label that is a string of characters that can be distributed on several lines to improve readability. The centre of the label must be placed on the centre of the container. The label may extend outside of the container.

**Auxiliary items:**

A *subunit* may carry auxiliary units, depending on its type.

A *macromolecule*, *nucleic acid feature*, or *complex subunit* can carry one or more *state variables* that add information about its state (Section 2.3.2). The state of such a *subunit* is defined as the set of all its *state variables*.

A *macromolecule*, *simple chemical*, *nucleic acid feature*, or *complex subunit* can carry one or more *units of information* (Section 2.3.1). These can characterize a domain, such as a binding site. Particular *units of information* are available for describing the material type (Section 2.2.1) and conceptual type (Section 2.2.2) of such a *subunit*.

| | | | |
|---|---|---|---|
|  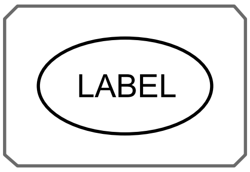 |  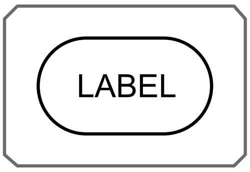 |  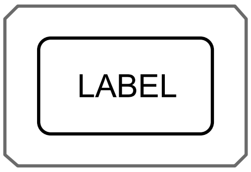 |  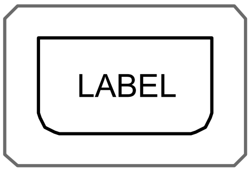 |
| *unspecified entity subunit* | *simple chemical subunit* | *macromolecule subunit* | *nucleic acid feature subunit* |
|  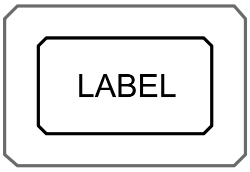 |  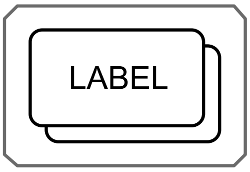 |  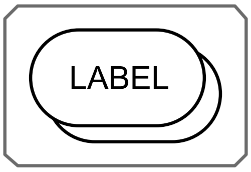 |  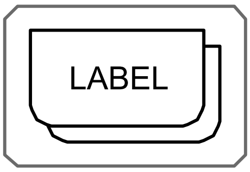 |
| *complex subunit* | *multimer of macromolecules subunit* | *multimer of simple chemicals subunit* | *multimer of nucleic acid feature subunit* |
|  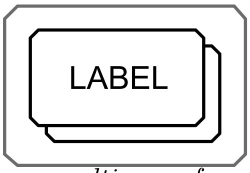 | | | |
| *multimer of complexes subunit* | | | |

**Table 2.7:** *The Process Description glyphs for the different types of* subunits. *Each* subunit *decorates a* complex.

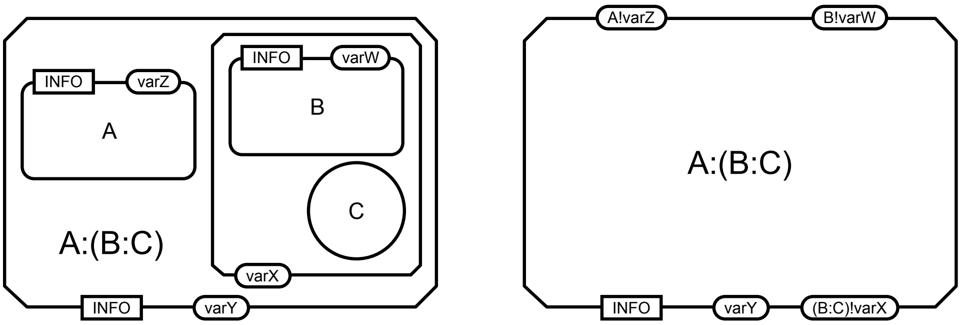

**Figure 2.8:** *Both these complex glyphs are equivalent. The complex on the left is described using* subunit *decorators. The complex on the right depicts the same information, without explicitly representing those subunits, that are only suggested by the label of the* complex. *However, their states are represented using* state variables *decorating the* complex.

#### 2.3.5 Glyph: *Submap terminal*

A *submap teminal* is a decorator of the *submap* (Section 2.13.1). It is a named handle, or reference, to both an *EPN* (Section 2.4) or *compartment* (Section 2.5.1) of the map, and a *tag* (Section 2.12.1) of the map the *submap* glyph refers to. Together with the *tag*, it allows linking glyphs of a map to their counterpart lying in a submap.

**SBO Term:**
Not applicable.

**Incoming arcs:**
One *equivalence arc* (Section 2.12.2).

**Outgoing arcs:**
None.

**Container:**
A *submap terminal* is represented by a rectangular shape fused to an empty arrowhead, as shown in Figure 2.9. The flat edge opposite to the arrowhead should be aligned to the edge of the *submap* glyph, and the incoming *equivalence arc* (Section 2.12.2) should be linked to its middle.

**Label:**
A *submap terminal* is identified by a label that is a string of characters that may be distributed on several lines to improve readability. The centre of the label must be placed on the centre of the container. The label may extend outside of the container.

**Auxiliary items:**
None.

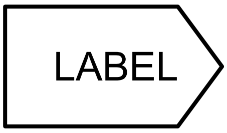

**Figure 2.9:** *The Process Description glyph for* submap terminal.

### 2.4 Entity pool nodes

An entity pool is a population of entities that cannot be distinguished from each other when it comes to the SBGN Process Description Level 1 map. For instance, all the molecular entities that fulfill the same role in a given process form an entity pool. As a result, an entity pool can represent different granularity levels, such as all the proteins, all the instances of a given protein, only certain forms of a given protein. To belong to a different compartment is sufficient to belong to different entity pools. Calcium ions in the endoplasmic reticulum and calcium ions in the cytosol belong to different entity pools when it comes to representing calcium release from the endoplasmic reticulum.

The Process Description contains six glyphs representing classes of material entities: *unspecified entity* (Section 2.4.1), *simple chemical* (Section 2.4.2), *macromolecule* (Section 2.4.3), *nucleic acid feature* (Section 2.4.4), *multimer* (Section 2.4.5) and *complex* (Section 2.4.6). (Specific types of macromolecules, such as protein, RNA, DNA, polysaccharide, and specific simple chemicals are not defined by Process Description but may be part of future levels of SBGN.) In addition to the material entities, Process Description represents two conceptual entity pools: *empty set* (Section 2.4.7), and *perturbing agent* (Section 2.4.8). Material and conceptual entities can optionally carry auxiliary units such as *units of information* (Section 2.3.1), *state variables* (Section 2.3.2) and *clone markers* (Section 2.3.3).

#### 2.4.1 Glyph: *Unspecified entity*

The simplest type of EPN is the *unspecified entity*: one whose type is unknown or simply not relevant to the purposes of the map. This arises, for example, when the existence of the entity has been inferred indirectly, or when the entity is merely a construct introduced for the needs of a map, without direct biological relevance. These are examples of situations where the *unspecified entity* glyph is appropriate. (Conversely, for cases where the identity of the entities composing the pool is known, there exist other, more specific glyphs described elsewhere in the specification.)

**SBO Term:**
SBO:0000285 ! material entity of unspecified nature

**Incoming arcs:**
Zero or more *production* arcs (Section 2.7.2).

**Outgoing arcs:**
Zero or more *consumption* arcs (Section 2.7.1), *modulation arcs* (Section 2.8), *logic arcs* (Section 2.10.1), or *equivalence arcs* (Section 2.12.2).

**Container:**
A *unspecified entity* is represented by an elliptic shape, as shown in Figure 2.10 on the next page. Note that the shape must remain an ellipse to avoid confusion with *simple chemical*, which is represented with a stadium shape (Section 2.4.2).

**Label:**
A *unspecified entity* is identified by a label that is a string of characters that may be distributed on several lines to improve readability. The centre of the label must be placed on the centre of the container. The label may extend outside of the container.

**Auxiliary items:**
An *unspecified entity* can carry a *clone marker* (see Section 2.3.3).

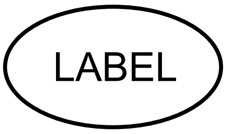

**Figure 2.10:** *The Process Description glyph for* unspecified entity*.*

#### 2.4.2 Glyph: *Simple chemical*

A simple chemical in SBGN is defined as the opposite of a macromolecule (Section 2.4.3): it is a chemical compound that is *not* formed by the covalent linking of pseudo-identical residues. Examples are an atom, a monoatomic ion, a salt, a radical, a solid metal, a crystal, etc.

**SBO Term:**

SBO:0000247 ! simple chemical

**Incoming arcs:**

Zero or more *production* arcs (Section 2.7.2).

**Outgoing arcs:**

Zero or more *consumption* arcs (Section 2.7.1), *modulation arcs* (Section 2.8), *logic arcs* (Section 2.10.1), or *equivalence arcs* (Section 2.12.2).

**Container:**

A *simple chemical* is represented by a "stadium" shape, that is two semicircles of the same radius joined by parallel line segments, as shown in Figure 2.11. If desired the parallel line segments can have zero length, and the shape is then identical to a circle. To avoid confusion with the *unspecified entity* (2.4.1), this form of the glyph must remain a circle and cannot be deformed into an ellipse.

**Label:**

A *simple chemical* is identified by a label that is a string of characters that may be distributed on several lines to improve readability. The centre of the label must be placed on the centre of the container.

**Auxiliary items:**

A *simple chemical* can carry one or more *units of information* (Section 2.3.1). Particular *units of information* are available for describing the material type (Section 2.2.1) and the conceptual type (Section 2.2.2) of a *simple chemical.*

A *simple chemical* can also carry a *simple clone marker* (see Section 2.3.3).

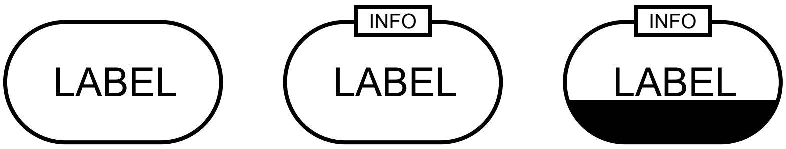

**Figure 2.11:** *The Process Description glyph for a* simple chemical*, shown plain and unadorned on the left, with an additional* unit of information *in the middle, and with a* simple clone marker *on the right.*

#### 2.4.3 Glyph: *Macromolecule*

Many biological processes involve *macromolecules*: biochemical substances that are built up from the covalent linking of pseudo-identical units. Examples of macromolecules include proteins, nucleic acids (RNA, DNA), and polysaccharides (glycogen, cellulose, starch, etc.). Attempting to define a separate glyph for all of these different molecules would lead to an explosion of symbols in SBGN, so instead, SBGN Process Description Level 1 specifies only one glyph for all macromolecules. The same glyph is to be used for a protein, a nucleic acid, a complex sugar, and so on. The exact nature of a particular macromolecule in a map is then clarified using its label and decorations, as it will become clearer below. (Future levels of SBGN may subclass the *macromolecule* and introduce different glyphs to differentiate between types of macromolecules.)

**SBO Term:**
SBO:0000245 ! macromolecule

**Incoming arcs:**
Zero or more *production* arcs (Section 2.7.2).

**Outgoing arcs:**
Zero or more *consumption* arcs (Section 2.7.1), *modulation arcs* (Section 2.8), *logic arcs* (Section 2.10.1), or *equivalence arcs* (Section 2.12.2).

**Container:**
A *macromolecule* is represented by a rectangular shape with rounded corners, as shown in Figure 2.12.

**Label:**
A *macromolecule* is identified by a label that is a string of characters that may be distributed on several lines to improve readability. The centre of the label must be placed on the centre of the container. The label may extend outside of the container.

**Auxiliary items:**
A *macromolecule* can carry one or more *state variables* that add information about its state (Section 2.3.2). The state of a *macromolecule* is defined as the set of all its *state variables*.

A *macromolecule* can also carry one or more *units of information* (Section 2.3.1). These can characterise a domain, such as a binding site. Particular *units of information* are available for describing the material type (Section 2.2.1) and the conceptual type (Section 2.2.2) of a *macromolecule*.

Finally, a *macromolecule* can also carry a *labelled clone marker* (see Section 2.3.3).

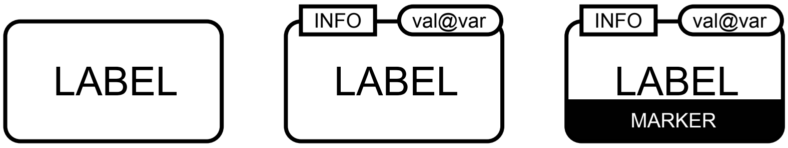

**Figure 2.12:** *The Process Description glyph for* macromolecule*, shown plain and unadorned on the left, with an additional* state variable *and a* unit of information *in the middle, and with a* labelled clone marker *on the right.*

#### 2.4.4 Glyph: *Nucleic acid feature*

The *nucleic acid feature* represents a fragment of a macromolecule carrying genetic information. A common use for this construct is to represent a gene or transcript. The label of this EPN and its *units of information* are often crucial for making the purpose clear to the reader of a map.

**SBO Term:**
SBO:0000354 ! informational molecule segment

**Incoming arcs:**

Zero or more *production* arcs (Section 2.7.2).

**Outgoing arcs:**

Zero or more *consumption* arcs (Section 2.7.1), *modulation arcs* (Section 2.8), *logic arcs* (Section 2.10.1), or *equivalence arcs* (Section 2.12.2).

**Container:**

A *nucleic acid feature* is represented by a rectangular shape whose bottom half has rounded corners. This design reminds us that we are fundamentally dealing with a unit of information carried by a macromolecule.

**Label:**

A *nucleic acid feature* is identified by a label that is a string of characters that may be distributed on several lines to improve readability. The centre of the label must be placed on the centre of the container. The label may extend outside of the container.

**Auxiliary items:**

A *nucleic acid feature* can carry one or more *state variables* that add information about its state (Section 2.3.2). The state of a *nucleic acid feature* is defined as the set of all its *state variables*.

A *nucleic acid feature* can also carry one or more *units of information* (Section 2.3.1). These can characterise a domain, such as a binding site. Particular *units of information* are available for describing the material type (Section 2.2.1) and the conceptual type (Section 2.2.2) of a *nucleic acid feature*.

Finally, a *nucleic acid feature* can also carry a *labelled clone marker* (see Section 2.3.3).

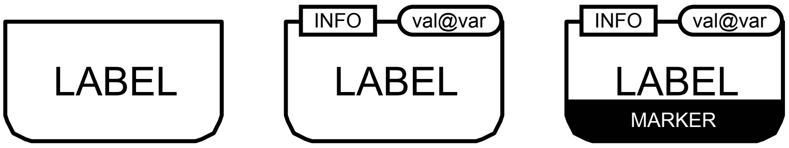

**Figure 2.13:** *The Process Description glyph for* nucleic acid feature*, shown plain and unadorned on the left, with an additional* state variable *and a* unit of information *in the middle, and with a* labelled clone marker *on the right.*

#### 2.4.5 Glyph: *Multimer*

As its name implies, a multimer is an aggregation of multiple identical or pseudo-identical entities held together by non-covalent bonds (thus, they are distinguished from polymers by the fact that the later involve covalent bonds). Here, *pseudo-identical* refers to the possibility that the entities differ chemically but retain some common global characteristic, such as a structure or function, and so can be considered identical within the context of the SBGN Process Description. An example of this is the homologous subunits in a hetero-oligomeric receptor. SBGN Process Description defines four different *multimer* glyphs: *simple chemical multimer*, *macromolecule multimer*, *nucleic acid feature multimer* and *complex multimer*.

**SBO Term:**

| | |
|---|---|
| | SBO:0000286 ! multimer |
| *Simple chemical multimer* | SBO:0000421 ! multimer of simple chemicals |
| *Macromolecule multimer* | SBO:0000420 ! multimer of macromolecules |
| *Complex multimer* | SBO:0000418 ! multimer of complexes |
| *Nucleic acid feature multimer* | SBO:0000419 ! multimer of informational molecule segments |

**Incoming arcs:**

Zero or more *production* arcs (Section 2.7.2).

**Outgoing arcs:**

Zero or more *consumption* arcs (Section 2.7.1), *modulation arcs* (Section 2.8), *logic arcs* (Section 2.10.1), or *equivalence arcs* (Section 2.12.2).

**Container:**

Each *multimer* is represented by a different shape depending on the bio-molecular nature of its pseudo-identical subunits, as shown in Table 2.8. The shape of a *multimer* consists of two *subunits* or *EPNs* shapes shifted horizontally and vertically, and stacked on top of another.

**Label:**

A *subunit* is identified by a label that is a string of characters that may be distributed on several lines to improve readability. The centre of the label must be placed on the centre of the shape. The label may extend outside of the shape. The label should refer to the pseudo-identical subunits, and not to the multimer itself.

**Auxiliary items:**

A *multimer* may carry auxiliary units, depending on its type.

A *macromolecule*, *nucleic acid feature*, or *complex multimer* can carry one or more *state variables* that add information about its state (Section 2.3.2). The state of such a *multimer* is defined as the set of all its *state variables.*

A *multimer* of any type can carry one or more *units of information* (Section 2.3.1). These can characterize a domain, such as a binding site. Particular *units of information* are available for describing the material type (Section 2.2.1), conceptual type (Section 2.2.2) and the cardinality (Section 2.2.5) of such a *multimer.*

Note that a *state variable* or a *unit of information* carried by a *multimer* actually applies to each of the subunits individually. If instead the *state variables* or the *units of information* are meant to apply to the whole multimeric assembly, a *macromolecule* (Section 2.4.3) or a *complex* (Section 2.4.6) should be used instead of a *multimer.* An assembly containing some *state variables* or *units of information* applicable to the subunits, and other *state variables* or *units of information* applicable to the assembly (for instance opening of a channel and phosphorylation of each of its subunits) should be represented by a *complex* (Section 2.4.6).

Finally, a *simple chemical multimer* can also carry a *simple clone marker* (Section 2.3.3), and a *macromolecule*, *nucleic acid feature* or *complex multimer* a *labelled clone marker* (Section 2.3.3).

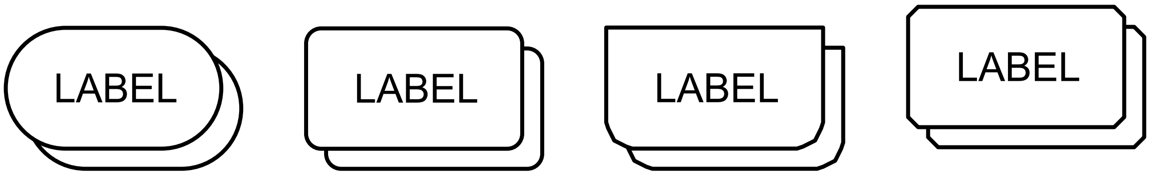

| *simple chemical multimer* | *macromolecule multimer* | *nucleic acid feature multimer* | *complex multimer* |
|---|---|---|---|

**Table 2.8:** *The Process Description glyphs for the different types of* multimers.

#### 2.4.6 Glyph: *Complex*

A *complex* represents a pool of biochemical entities, each composed of other biochemical entities, whether macromolecules, simple chemicals, multimers, or other complexes. The resulting entity may have its own identity, properties and function in an SBGN map. The *complex* can be described by the set of *subunits* (Section 2.3.4) it contains (see Figure 2.8 on page 14). This description is entirely optional and is there to assist the user with a visual shorthand about the composition of the complex.

**SBO Term:**

SBO:0000253 ! non-covalent complex

**Incoming arcs:**

Zero or more *production* arcs (Section 2.7.2).

**Outgoing arcs:**

Zero or more *consumption* arcs (Section 2.7.1), *modulation arcs* (Section 2.8), *logic arcs* (Section 2.10.1), or *equivalence arcs* (Section 2.12.2).

**Container:**

A *complex* is represented by a rectangular shape with cut-corners (that is, an octogonal shape with sides of two different lengths). If the *complex* is described by a set of *subunits*, then its shape should surround those of its *subunits*, and the size of the cut-corners should be adjusted so that there is no overlap between its shape and those of its *subunits*. The shapes of the *subunits* must not overlap.

**Label:**

A *complex* is identified by a label that is a string of characters that may be distributed on several lines to improve readability. In the case where the *complex* is not described by a set of *subunits*, the centre of the label must be placed on the centre of the *complex*'s shape. In the case where the *complex* is described by a set of *subunits*, the label may be positioned to optimize the clarity and avoid overlapping, ideally between the bottom-most or the upper-most *subunit* and the border of the *complex*.

**Auxiliary items:**

A *complex* can carry one or more *state variables* that add information about its state (Section 2.3.2). .

A *complex* can also carry one or more *units of information* (Section 2.3.1). These can characterise a domain, such as a binding site. Particular *units of information* are available for describing the material type (Section 2.2.1) and the conceptual type (Section 2.2.2) of a *complex*.

Finally, a *complex* can also carry a *labelled clone marker* (see Section 2.3.3).

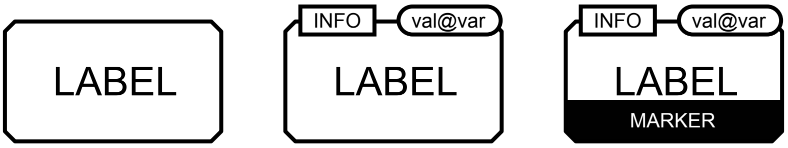

**Figure 2.14:** *The Process Description glyph for* complex*, shown plain and unadorned on the left, with an additional* state variable *and a* unit of information *in the middle, and with a* labelled clone marker *on the right.*

#### 2.4.7 Glyph: *Empty Set*

It is useful to have the ability to represent the creation of an entity or a state from an unspecified source, that is, from something that one does not need or wish to make precise. For instance, in a model where the production of a protein is represented, it may not be desirable to represent all of the amino acids, sugars and other metabolites used, or the energy involved in the protein's creation. Similarly, we may not wish to bother representing the details of the destruction or decomposition of some biochemical species into a large number of more primitive entities, preferring instead to simply say that the species "disappears into a sink". Yet another example is that one may need to represent an input (respectively, output) into (respectively, from) a compartment without explicitly representing a transport process from a source (respectively, to a target).

For these and other situations, SBGN defines a single glyph representing the involvement of an external pool of entities. The symbol used in SBGN is borrowed from the mathematical symbol for "empty set", but it is important to note that it does not actually represent a true absence of everything or a physical void—it represents the absence of the corresponding structures in the model, that is, the fact that the external pool is conceptually outside the scope of the map.

A frequently asked question is, why bother having an explicit symbol at all? The reason is that one cannot simply use an arc that does not terminate on a node, because the dangling end could be mistaken to be pointing to another node in the map. This is specially true if the map is rescaled, causing the spacing of elements in the map to change. The availability and use of an explicit symbol for sources and sinks is crucial.

**SBO Term:**
SBO:0000291 ! empty set

**Incoming arcs:**
Zero or one *production* arcs (Section 2.7.2).

**Outgoing arcs:**
Zero or one *consumption* arcs (Section 2.7.1).

**Container:**
An *empty set* is represented by a circular shape crossed by a bar linking the lower-left and upper-right corners of the circle's bounding box, as shown in Figure 2.15.

**Label:**
None.

**Auxiliary items:**
None.

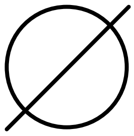

**Figure 2.15:** *The Process Description glyph for* empty set.

#### 2.4.8 Glyph: *Perturbing agent*

Biochemical networks can be affected by external influences. Those influences can be the effect of well-defined physical perturbing agents, such as a light pulse or a change in temperature; they can also be more complex and not well-defined phenomena, for instance the outcome of a biological process, an experimental setup, or a mutation. For these situations, Process Description provides the *perturbing agent* glyph. It is an EPN, and represents the amount of perturbing agent applied to a process.

**SBO Term:**
SBO:0000405 ! perturbing agent

**Incoming arcs:**
None.

**Outgoing arcs:**
One or more *modulation arcs* (Section 2.8) or *logic arcs* (Section 2.10.1), zero or more *equivalence arcs* (Section 2.12.2).

**Container:**
A *perturbing agent* is represented by a by a modified hexagonal shape having two opposite concave faces, as shown in Figure 2.16.

**Label:**
A *perturbing agent* is identified by a label that is a string of characters that may be distributed on several lines to improve readability. The centre of the label must be placed on the centre of the container. The label may extend outside of the container.

**Auxiliary items:**
A *perturbing agent* can carry one or more *units of information* (Section 2.3.1). Particular *units of information* are available for describing the material type (Section 2.2.1) and the conceptual type (Section 2.2.2) of a *perturbing agent*, as well as its physical characteristic (see Section 2.2.4).

A *perturbing agent* can also carry a *simple clone marker* (see Section 2.3.3).

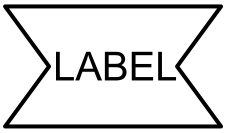

**Figure 2.16:** *The Process Description glyph for* perturbing agent.

#### 2.4.9 Examples of complex EPNs

In this section, we provide examples of Entity Pool Node representations drawn using the SBGN Process Description Level 1 glyphs described above.

Figure 2.17 represents a pool of calcium/calmodulin kinase II entities, each with phosphorylation on the sites threonine 286 and 306, as well as catalytic and autoinhibitory domains. Note the use of *units of information* and *state variables*.

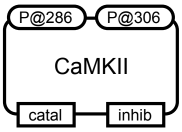

**Figure 2.17:** *An example representation of calcium/calmodulin kinase II EPN.*

Figure 2.18 represents the glutamate receptor in the open state, with both phosphorylation and glycosylation. The entity carries two functional domains, the ligand-binding domain and the ion pore, and its chemical nature is presided.

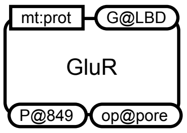

**Figure 2.18:** *An example of a glutamate receptor in the open state.*

### 2.5 Defined sets of entity pool nodes

#### 2.5.1 Glyph: *Compartment*

A compartment is a logical or physical structure that contains entity pool nodes. An EPN can only belong to one compartment. Therefore, the "same" biochemical species located in two different compartments are in fact two different pools.

**SBO Term:**
SBO:0000290 ! physical compartment

**Incoming arcs:**
None.

**Outgoing arcs:**
Zero or more *equivalence arcs* (Section 2.12.2).

**Container:**
A *compartment* is represented by a surface enclosed in a continuous border or located between continuous borders. These borders should be noticeably thicker than the borders of the EPNs. A compartment can take **any** shape. A compartment must always be entirely enclosed.

**Label:**
A *compartment* is identified by a label that is a string of characters that may be distributed on several lines to improve readability. The label can be placed anywhere in the shape. The label may extend outside of the shape.

**Auxiliary items:**
A *compartment* can carry one or more *units of information* (Section 2.3.1). These can characterise the physical environment, such as pH, temperature or voltage.

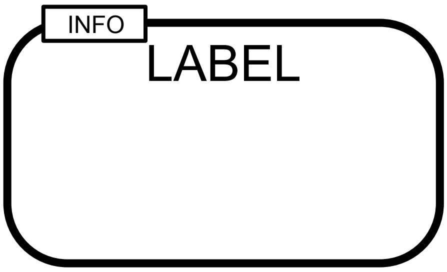

**Figure 2.19:** *The Process Description glyph for* compartment.

To allow more aesthetically pleasing and understandable maps, compartments are allowed to overlap each other visually, but it must be kept in mind that this does not mean the top compartment contains part of the bottom compartment. Figure 2.20 shows two semantically equivalent placement of compartments:

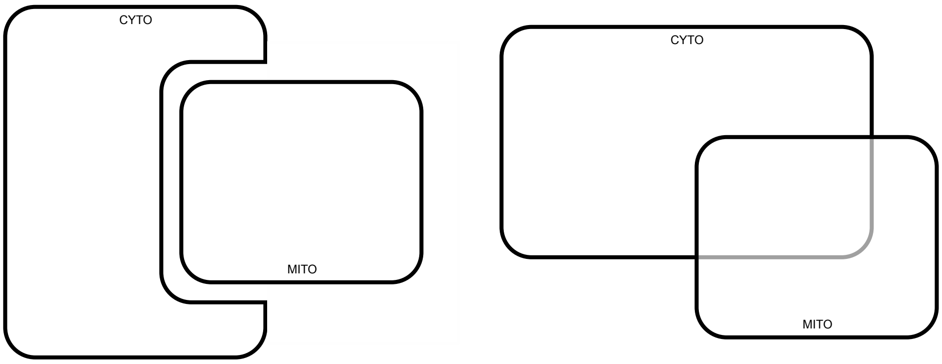

**Figure 2.20:** *Overlapped compartments are permitted, but the overlap does not imply containment.*

Overlapped (hidden) part of the compartment should not contain any object which could be covered by an overlapping compartment. Figure 2.21 illustrates the problem using an incorrect map.

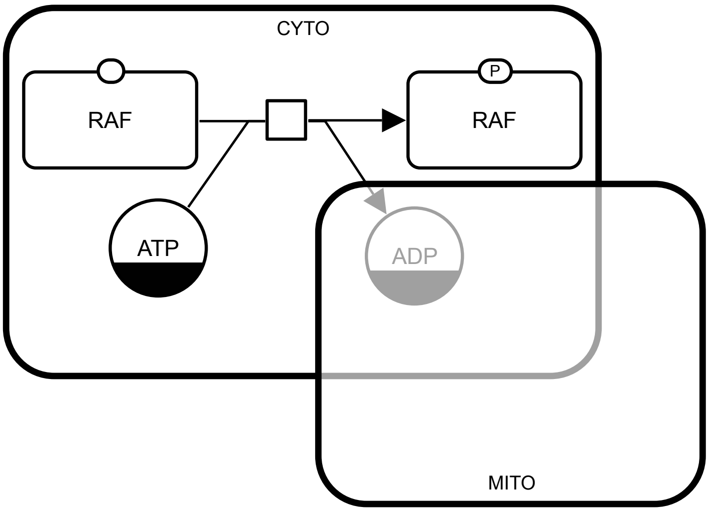

**Figure 2.21:** *Example of an* ***incorrect*** *map. Overlapped compartments must not obscure other objects.*

### 2.6 Process nodes

Process nodes represent processes that transform one or several entity pools into one or several entity pools, identical or different. SBGN Process Description Level 1 defines a generic *process* (Section 2.6.1), as well as five more specific ones: the *omitted process* (Section 2.6.2), the *uncertain process* (Section 2.6.3), the *association* (Section 2.6.4), the *dissociation* (Section 2.6.5), and the *phenotype* (Section 2.6.6). In future levels of the SBGN Process Description language, more processes may be defined. (One can even envision the development of a controlled vocabulary of processes, as is done now for *EPNs*; see Section 2.2.)

#### 2.6.1 Glyph: *Process*

A *process* represents a generic process that transforms a set of entity pools (represented by *EPNs* in SBGN Process Description Level 1) into another set of entity pools.

**SBO Term:**

SBO:0000375 ! process

**Incoming arcs:**

One ore more *consumption* arcs (Section 2.7.1)[1], zero or more *modulation* arcs (Section 2.8).

**Outgoing arcs:**

One or more *production* arcs (Section 2.7.2).

**Container:**

A *process* is represented by a square shape. The shape is linked to two ports, that are small arcs attached to the centres of opposite sides of the shape, as shown in Figure 2.22. The incoming *consumption* (Section 2.7.1) and outgoing *production* (Section 2.7.2) arcs are linked to the extremities of those ports.

The *modulation arcs* (Section 2.8) point to the other two sides of the shape.

**Label:**

None.

**Auxiliary items:**

None.

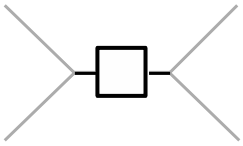

**Figure 2.22:** *The Process Description glyph for* process.

A *process* is the basic process node in Process Description. It represents a process that transforms a given set of biochemical entities—macromolecules, simple chemicals or unspecified entities—into another set of biochemical entities. Such a transformation might imply modification of covalent bonds (conversion), modification of the relative position of constituents (conformational process) or movement from one compartment to another (translocation).

A cardinality label may be associated with *consumption* (Section 2.7.1) or *production* (Section 2.7.2) arcs to indicate the stoichiometry of the process. This label becomes a requirement when the exact composition of the number of copies of the inputs or outputs to a reaction are ambiguous in the map.

A process is regarded as reversible if both ports of the process are connected to *production* arcs (see section 3.5.2.5).

The example in Figure 2.23 on the next page illustrates the use of a *process* node to represent the phosphorylation of a protein in a Process Description.

[1]Zero *consumption arcs* are allowed in the case of a reversible process.

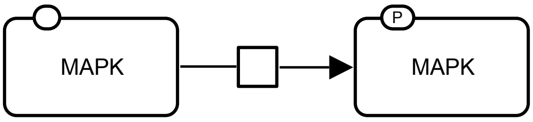

**Figure 2.23:** *Phosphorylation of the protein MAP kinase.*

The example in Figure 2.24 illustrates the use of a *process* node to represent a reaction between two reactants that generates three products.

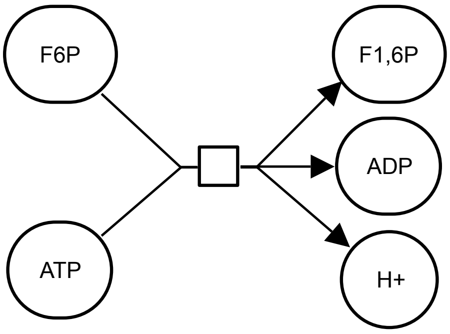

**Figure 2.24:** *Reaction between ATP and fructose-6-phosphate to produce fructose-1,6-biphosphate, ADP and a proton.*

The example in Figure 2.25 illustrates the use of a *process* node to represent a translocation. The large round-cornered rectangle represents a compartment border (see Section 2.5.1).

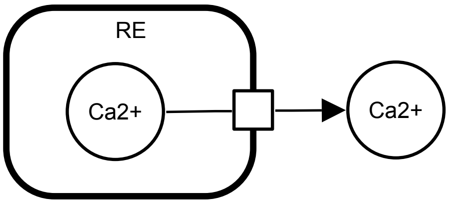

**Figure 2.25:** *Translocation of calcium ion out of the endoplasmic reticulum. Note that the* process *does not have to be located on the boundary of the* compartment*. A* process *is not attached to any* compartment*.*

The example in Figure 2.26 illustrates the use of a *process* node to represent the reversible opening and closing of an ionic channel in a Process Description.

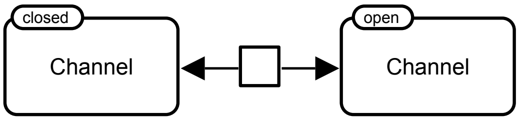

**Figure 2.26:** *Reversible opening and closing of an ionic channel.*

When such a reversible process is asymmetrically modulated, it must be represented by two different *processes* in a Process Description. Figure 2.27 on the next page illustrates the use of two *process* nodes to represent the reversible activation of a G-protein coupled receptor. In the absence of any effector, an equilibrium exists between the inactive and active forms. The agonist stabilises the active form, while the inverse agonist stabilises the inactive form.

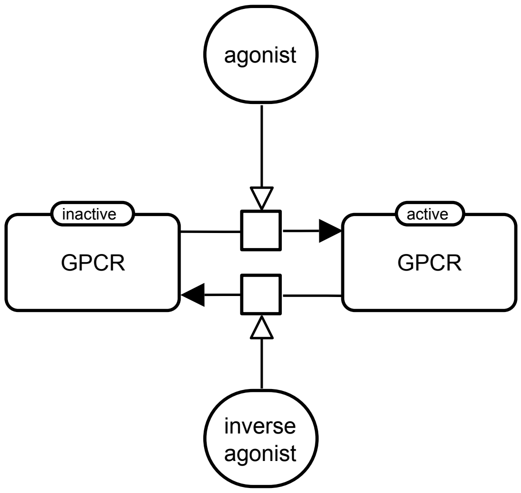

**Figure 2.27:** *The reversible activation of a G-protein coupled receptor.*

The example in Figure 2.28 presents the conversion of two galactoses into a lactose. Galactoses are represented by only one *simple chemical*, the cardinality being carried by the *consumption* arc.

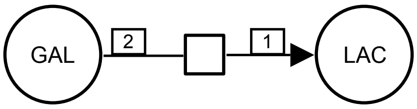

**Figure 2.28:** *Conversion of two galactoses into a lactose.*

#### 2.6.2 Glyph: *Omitted process*

Omitted processes are processes that are known to exist, but are omitted from the map for the sake of clarity or parsimony. A single *omitted process* can represent any number of actual processes. The *omitted process* is different from a *submap*. While a *submap* references to an explicit content, that is hidden in the main map, the *omitted process* does not "hide" anything within the context of the map, and cannot be "unfolded".

**SBO Term:**
SBO:0000397 ! omitted process

**Incoming arcs:**
One ore more *consumption* arcs (Section 2.7.1)[2], zero or more *modulation* arcs (Section 2.8).

**Outgoing arcs:**
One or more *production* arcs (Section 2.7.2).

**Container:**
An *omitted process* is represented by a square shape that contains two parallel slanted lines oriented northwest-to-southeast and separated by an empty space. The shape is

[2]Zero *consumption arcs* are allowed in the case of a reversible process.

linked to two ports, that are small arcs attached to the centres of opposite sides of the shape, as shown in Figure 2.29. The incoming *consumption* (Section 2.7.1) and outgoing *production* (Section 2.7.2) arcs are linked to the extremities of those ports.

The *modulation arcs* (Section 2.8) point to the other two sides of the shape.

**Label:**
None.

**Auxiliary items:**
None.

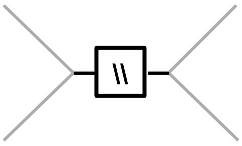

**Figure 2.29:** *The Process Description glyph for* omitted process*.*

#### 2.6.3 Glyph: *Uncertain process*

Uncertain processes are processes that may not exist. A single *uncertain process* can represent any number of actual processes.

**SBO Term:**
SBO:0000396 ! uncertain process

**Incoming arcs:**
One ore more *consumption* arcs (Section 2.7.1)[3], zero or more *modulation* arcs (Section 2.8).

**Outgoing arcs:**
One or more *production* arcs (Section 2.7.2).

**Container:**
A *process* is represented by a square shape containing a question mark. The shape is linked to two ports, that are small arcs attached to the centres of opposite sides of the shape, as shown in Figure 2.30. The incoming *consumption* (Section 2.7.1) and outgoing *production* (Section 2.7.2) arcs are linked to the extremities of those ports.

The *modulation arcs* (Section 2.8) point to the other two sides of the shape.

**Label:**
None.

**Auxiliary items:**
None.

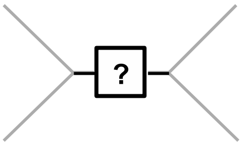

**Figure 2.30:** *The Process Description glyph for an* uncertain process*.*

[3]Zero *consumption arcs* are allowed in the case of a reversible process.

#### 2.6.4 Glyph: *Association*

The *association* between one or more *EPNs* represents the non-covalent binding of the biological entities represented by those *EPNs* into a larger complex.

**SBO Term:**
SBO:0000177 ! non-covalent binding

**Incoming arcs:**
One ore more *consumption* arcs (Section 2.7.1), zero or more *modulation* arcs (Section 2.8).

**Outgoing arcs:**
One *production* arc (Section 2.7.2).

**Container:**
An *association* is represented by a circular filled shape. The shape is linked to two ports, that are small arcs attached to the centres of opposite sides of the shape, as shown in Figure 2.31. The incoming *consumption* (Section 2.7.1) and outgoing *production* (Section 2.7.2) arcs are linked to the extremities of those ports.

The *modulation arcs* (Section 2.8) point to the other two sides of the shape.

**Label:**
None.

**Auxiliary items:**
None.

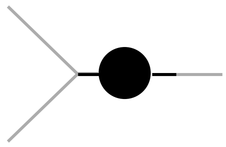

**Figure 2.31:** *The Process Description glyph for* association.

The example in Figure 2.32 illustrates the association of cyclin and CDC2 kinase into the Maturation Promoting Factor.

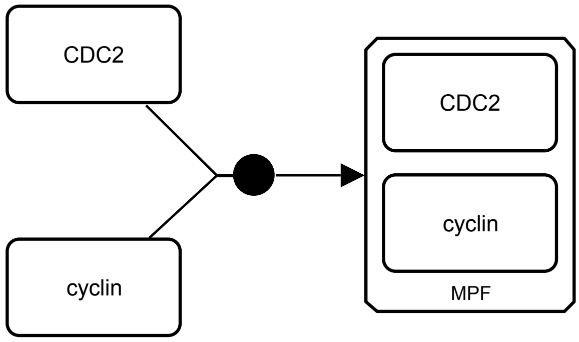

**Figure 2.32:** *Association of cyclin and CDC2 kinase into the Maturation Promoting Factor.*

Figure 2.33 on the next page gives an example illustrating the association of a pentameric macromolecule (a nicotinic acetylcholine receptor) with a simple chemical (the local anesthetic chlorpromazin) in an unnamed complex.

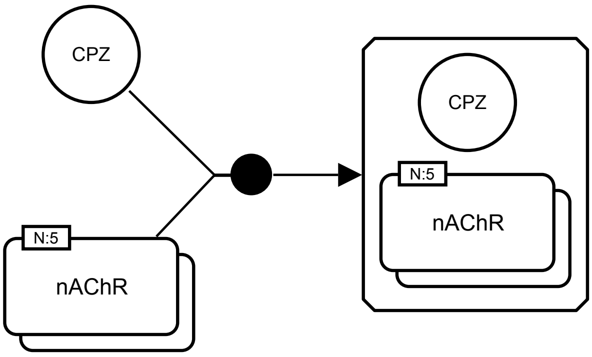

**Figure 2.33:** *The association of a pentameric macromolecule with a simple chemical in an unnamed complex.*

An association does not necessarily result in the formation of a *complex*; it can also produce a *multimer*, or a *macromolecule* (although the latter case is semantically borderline). Figure 2.34 gives an example of this, using the formation of hemoglobin.

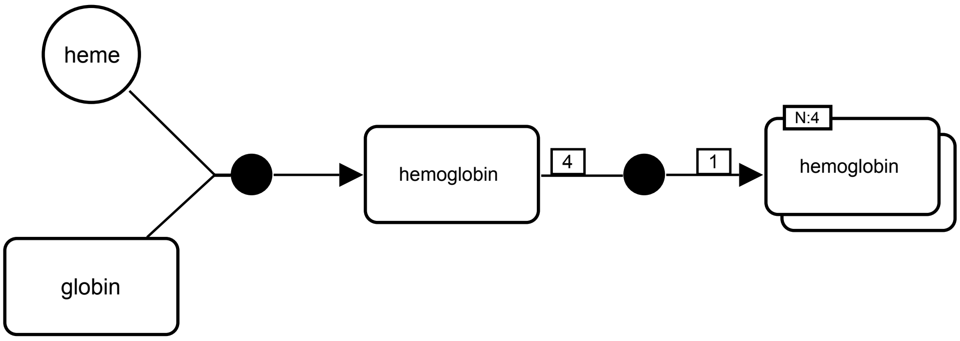

**Figure 2.34:** *Formation of hemoglobin.*

#### 2.6.5 Glyph: *Dissociation*

The *dissociation* of an *EPN* into one or more *EPNs* represents the rupture of a non-covalent binding between the biological entities represented by those *EPNs*.

**SBO Term:**

SBO:0000180 ! dissociation

**Incoming arcs:**

One *consumption* arc (Section 2.7.1), zero or more *modulation* arcs (Section 2.8).

**Outgoing arcs:**

One or more *production* arcs (Section 2.7.2).

**Container:**

A *process* is represented by a circular shape containing another concentric circle. The shape is linked to two ports, that are small arcs attached to the centres of opposite sides of the shape, as shown in Figure 2.35 on the next page. The incoming *consumption*

(Section 2.7.1) and outgoing *production* (Section 2.7.2) arcs are linked to the extremities of those ports.

The *modulation arcs* (Section 2.8) point to the other two sides of the shape.

**Label:**

None.

**Auxiliary items:**

None.

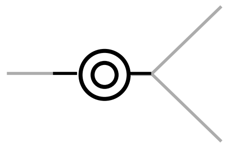

**Figure 2.35:** *The Process Description glyph for* dissociation.

The example in Figure 2.36 illustrates the dissociation of the small and large ribosomal subunits from a messenger RNA.

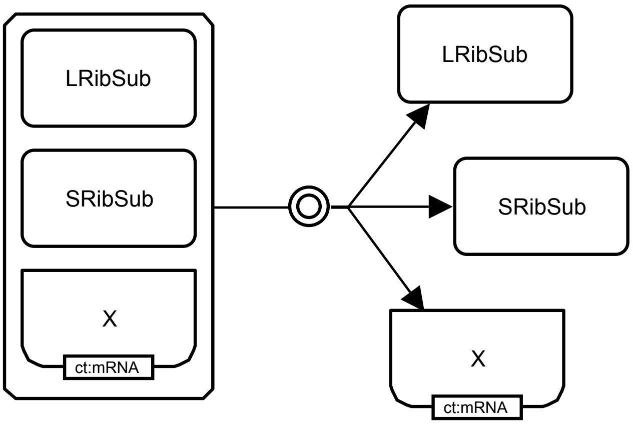

**Figure 2.36:** *Dissociation of the small and large ribosomal subunits from a messenger RNA.*

#### 2.6.6 Glyph: *Phenotype*

A biochemical network can generate phenotypes or affect biological processes. Such processes can take place at different levels and are independent of the biochemical network itself. To represent these processes in a map, Process Description defines the *phenotype* glyph.

**SBO Term:**

SBO:0000358 ! phenotype

**Incoming arcs:**

One or more *modulation* arcs (Section 2.8).

**Outgoing arcs:**

None.

**Container:**

A *phenotype* is represented by an elongated hexagonal shape, as shown in Figure 2.37 on the next page.

**Label:**

A *phenotype* is identified by a label that is a string of characters that may be distributed on several lines to improve readability. The centre of the label must be placed on the centre of the shape. The label may extend outside of the shape.

**Auxiliary items:**

None.

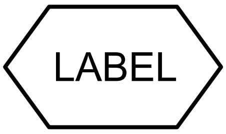

**Figure 2.37:** *The Process Description glyph for* phenotype.

### 2.7 Flux arcs

Processes transform entity pools into other entity pools (Section 2.6). Flux arcs allow representing which entity pools are consumed and produced by a process. *Consumption* arcs link processes to their reactants, and *production* arcs link processes to their products. SBGN Process Description Level 1 does not provide any specific arc to represent the fluxes of a reversible process, that can be conveniently represented using only *production* arcs.

#### 2.7.1 Glyph: *Consumption*

*Consumption* is the arc used to represent the fact that an entity pool is consumed by a process, but is not produced by the process.

**SBO Term:**

SBO:0000394 ! consumption

**Origin:**

One *EPN* (Section 2.4).

**Target:**

One *process node* (Section 2.6).

**Symbol:**

No particular symbol is used to represent a *consumption*, as shown in Figure 2.38.

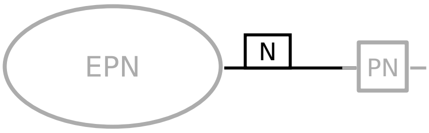

**Figure 2.38:** *The Process Description glyph for* consumption.

A cardinality label may be associated with *consumption* (Section 2.7.1) or *production* (Section 2.7.2) arcs, indicating the stoichiometry of a process. This label is a number enclosed in a rectangular container with one of the long sides adjacent to the *consumption* arc. The cardinality is required to eliminate ambiguity when the exact composition, or the number of copies, of the inputs or outputs to a reaction are ambiguous from the map. An example is a multimer of six subunits dissociating into two monomers and two dimers. Without stoichiometry labels another result, such as four monomers and one dimer could be inferred. Once assigned to one arc connecting to a process node, cardinality should be represented on all *consumption* and *production* arcs connected to that process node to avoid misinterpretation.

Omitted cardinality on one edge only should not be treated as cardinality of one, but as an unspecified cardinality. In most cases, the exact value may be derived from the context, but unless cardinality is explicitly shown, it should be considered as unspecified. In the case where the stoichiometry of some part of the process is not known, or undefined, a question mark (”?”) should be used within the cardinality label of the corresponding arcs.

#### 2.7.2 Glyph: *Production*

*Production* is the arc used to represent the fact that an entity pool is produced by a process. In the case of a reversible process, the *production* arc represents both a consumption and a production.

**SBO Term:**

SBO:0000393 ! production

**Origin:**

One *process node* (Section 2.6).

**Target:**

One *EPN* (Section 2.4).

**Symbol:**

The target extremity of a *production* carries a filled arrowhead, as shown in Figure 2.39.

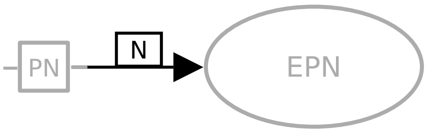

**Figure 2.39:** *The Process Description glyph for* production.

A cardinality label may be associated with a *production* arc, indicating the stoichiometry of a process.

Figure 2.40 on the following page illustrates the use of *consumption/production* arc cardinality labels to represent the stoichiometry of a process.

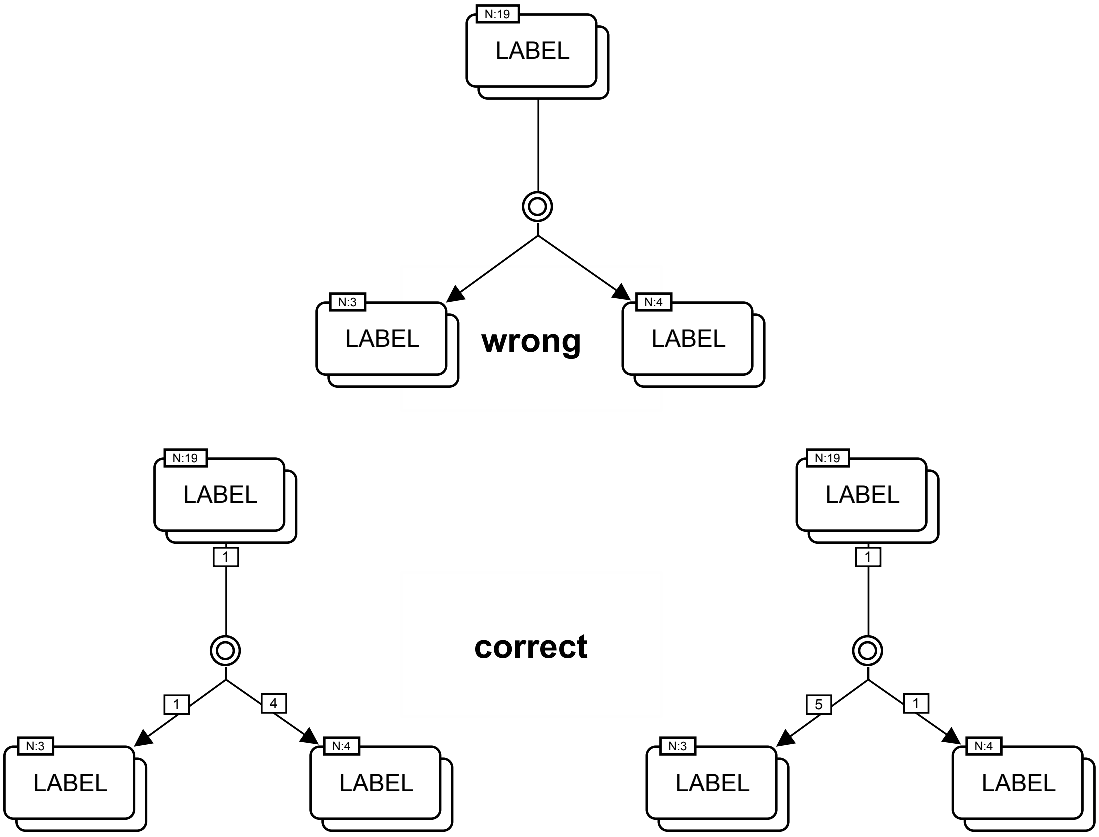

**Figure 2.40:** *Cardinality for production arcs. The process on the top is wrong as the stoichiometry is not represented, which leads to ambiguity.*

### 2.8 Modulation arcs

Modulation arcs represent influences of entity pools on processes. A stimulation affects positively the flux of a process, while an inhibition affects it negatively. SBGN Process Description Level 1 provides six modulation arcs: *modulation*, *stimulation*, *catalysis*, *necessary stimulation* and *inhibition*.

#### 2.8.1 Glyph: *Modulation*

A general modulation where the exact nature of the modulation is not specified or not known. The *modulation* glyph can be used when one does not know the precise direction of the effect.

**SBO Term:**
SBO:0000168 ! control

**Origin:**
One *EPN* (Section 2.4) or *logical operator* (Section 2.9).

**Target:**
One *process node* (Section 2.6).

**Symbol:**
The target extremity of a *modulation* carries an empty diamond, as shown in Figure 2.41 on the next page.

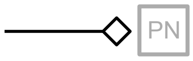

**Figure 2.41:** *The Process Description glyph for* modulation.

Figure 2.42 represents the effect of nicotine on the process between closed and open states of a nicotinic acetylcholine receptor. High concentrations of nicotine open the receptor while low concentrations can desensitize it without opening.

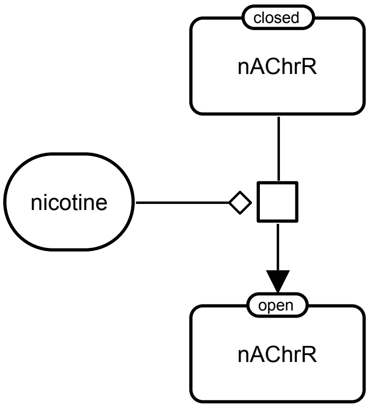

**Figure 2.42:** *Modulation of nicotinic receptor opening by nicotine.*

#### 2.8.2 Glyph: *Stimulation*

A stimulation affects **positively** the flux of a process represented by the target process. This stimulation can be, for instance, a catalysis or a positive allosteric regulation. Note that *catalysis* exists independently in SBGN, see Section 2.8.3.

**SBO Term:**

SBO:0000170 ! stimulation

**Origin:**

One *EPN* (Section 2.4) or *logical operator* (Section 2.9).

**Target:**

One *process node* (Section 2.6).

**Symbol:**

The target extremity of a *stimulation* carries an empty arrowhead, as shown in Figure 2.43.

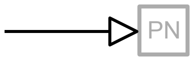

**Figure 2.43:** *The Process Description glyph for* stimulation.

#### 2.8.3 Glyph: *Catalysis*

A catalysis is a particular case of stimulation, where the effector affects positively the flux of a process represented by the target process. The positive effect on the process is due to the lowering of the activation energy of a reaction.

**SBO Term:**
SBO:0000172 ! catalysis

**Origin:**
One *EPN* (Section 2.4) or *logical operator* (Section 2.9).

**Target:**
One *process node* (Section 2.6).

**Symbol:**
The target extremity of a *catalysis* carries an empty circle, as shown in Figure 2.44.

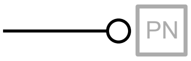

**Figure 2.44:** *The Process Description glyph for* catalysis*.*

#### 2.8.4 Glyph: *Inhibition*

An inhibition **negatively** affects the flux of a process represented by the target process. This inhibition can be, for instance, a competitive inhibition or an allosteric inhibition.

**SBO Term:**
SBO:0000169 ! inhibition

**Origin:**
One *EPN* (Section 2.4) or *logical operator* (Section 2.9).

**Target:**
One *process node* (Section 2.6).

**Symbol:**
The target extremity of an *inhibition* carries a bar perpendicular to the arc, as shown in Figure 2.45.

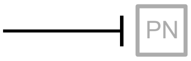

**Figure 2.45:** *The Process Description glyph for* inhibition*.*

#### 2.8.5 Glyph: *Necessary stimulation*

A necessary stimulation is one that is necessary for a process to take place. A process modulated by a necessary stimulation can only occur when this necessary stimulation is active.

**SBO Term:**
SBO:0000171 ! necessary stimulation

**Origin:**
One *EPN* (Section 2.4) or *logical operator* (Section 2.9).

**Target:**
One *process node* (Section 2.6).

**Symbol:**

The target extremity of a *necessary stimulation* carries an open arrowhead (to remind that it is a *stimulation*) coming after a larger perpendicular bar, as shown in Figure 2.46.

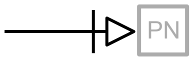

**Figure 2.46:** *The Process Description glyph for* Necessary Stimulation*.*

The example in Figure 2.47 below describes the transcription of a gene X, that is the creation of a messenger RNA X triggered by the gene X. The creation of the protein X is then triggered by the mRNA X. (Note that the same example could be represented using the gene as reactant and product, although it is semantically different.)

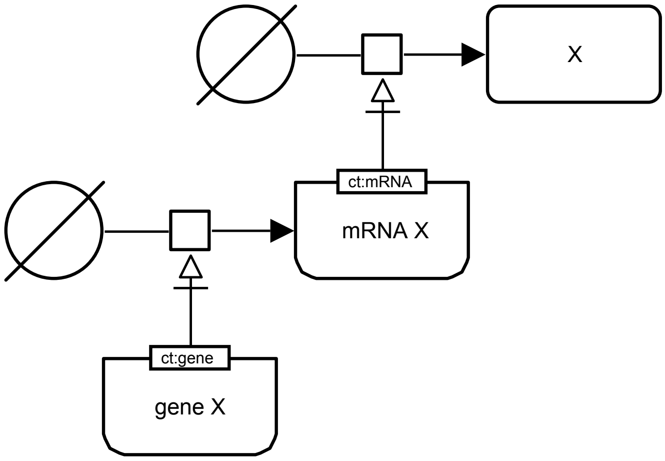

**Figure 2.47:** *The creation of a messenger RNA X triggered by the gene X.*

The example in Figure 2.48 on the following page below describes the transport of calcium ions out of the endoplasmic reticulum. Without IP3 receptor, there is no calcium flux, therefore, one cannot use a *stimulation*. The *necessary stimulation* instead represents the necessity for the receptor to be present for the transport to take place.

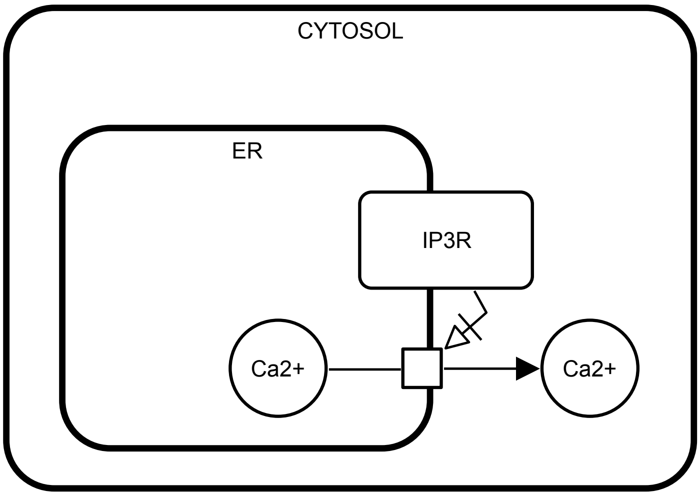

**Figure 2.48:** *The transport of calcium ions out of the endoplasmic reticulum into the cytosol. Note that IP3R crosses both compartment boundaries. This is allowed, but the Macromolecule should only belong to one of the compartments see section D.1 for more discussion of this issue.*

### 2.9 Logical operators

The *logical operator* performs an operation on one or more inputs to give a unique output. The *and*, *or*, and *not* operators perform a Boolean operation to give a binary output, while the *equivalence* operator performs a union of pools to give a new pool.

#### 2.9.1 Glyph: *And*

The output of an *and* glyph is True if all its inputs are True, and False otherwise.

**SBO Term:**
SBO:0000173 ! and

**Incoming arcs:**
One or more *logic arcs* (Section 2.10.1).

**Outgoing arcs:**
One *logic arc* (Section 2.10.1) or *modulation arc* (Section 2.8).

**Container:**
An *and* operator is represented by a circular shape containing the word "AND". The shape is linked to two ports, that are small arcs attached to the centres of opposite sides of the shape, as shown in Figure 2.49 on the next page. The incoming *logic arcs* (Section 2.10.1) are linked to the extremity of the leftmost or uppermost port, while the outgoing *logic arc* (Section 2.10.1) or *modulation* (Section 2.8.1) is linked to the extremity of the rightmost or bottommost port.

**Label:**
None.

**Auxiliary items:**
None.

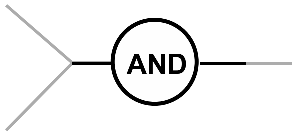

**Figure 2.49:** *The Process Description glyph for* and. *Only two inputs are represented, but more would be allowed.*

#### 2.9.2 Glyph: *Or*

The output of an *or* glyph is True if at least one of its inputs is True, and False otherwise.

**SBO Term:**
SBO:0000174 ! or

**Incoming arcs:**
One or more *logic arcs* (Section 2.10.1).

**Outgoing arcs:**
One *logic arc* (Section 2.10.1) or *modulation arc* (Section 2.8).

**Container:**
An *or* operator is represented by a circular shape containing the word "OR". The shape is linked to two ports, that are small arcs attached to the centres of opposite sides of the shape, as shown in Figure 2.50. The incoming *logic arcs* (Section 2.10.1) are linked to the extremity of the leftmost or uppermost port, while the outgoing *logic arc* (Section 2.10.1) or *modulation* (Section 2.8.1) is linked to the extremity of the rightmost or bottommost port.

**Label:**
None.

**Auxiliary items:**
None.

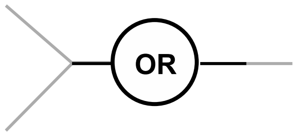

**Figure 2.50:** *The Process Description glyph for* or. *Only two inputs are represented, but more would be allowed.*

#### 2.9.3 Glyph: *Not*

The output of a *not* glyph is True if its input is False, and False otherwise.

**SBO Term:**
SBO:0000238 ! not

**Incoming arcs:**
One *logic arc* (Section 2.10.1).

**Outgoing arcs:**
One *logic arc* (Section 2.10.1) or *modulation arc* (Section 2.8).

**Container:**

A *not* operator is represented by a circular shape containing the word "NOT". The shape is linked to two ports, that are small arcs attached to the centres of opposite sides of the shape, as shown in Figure 2.51. The incoming *logic arc* (Section 2.10.1) is linked to the extremity of the leftmost or uppermost port, while the outgoing *logic arc* (Section 2.10.1) or *modulation* (Section 2.8.1) is linked to the extremity of the rightmost or bottommost port.

**Label:**

None.

**Auxiliary items:**

None.

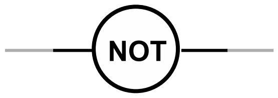

**Figure 2.51:** *The Process Description glyph for* not.

#### 2.9.4 Glyph: *Equivalence*

The *equivalence operator* provides a mechanism to sub-class EPNs. For example, this operator allows specifying that macromolecules or nucleic acid features $X_1$, $X_2$, and $X_3$ are subclasses of X. This avoids the need for processes that apply to all subtypes of X to be duplicated and simplifies cases where combinatorial explosions of the number of EPNs or PNs may arise. Caution should be used with this glyph as there is the possibility that ambiguity may be introduced into a SBGN map. Examples of the correct usage of this glyph are provided in Appendix B together with examples of misuse along with a proposed set of guidelines for the use of this *equivalence operator*.

**SBO Term:**

SBO:0000392 ! equivalence

**Incoming arcs:**

Two or more *logic arcs* (Section 2.10.1).

**Outgoing arcs:**

One *logic arc* (Section 2.10.1).

**Container:**

An *equivalence* operator is represented by a circular shape containing the symbol "Ξ" (letter "xi" of the Greek alphabet). The choice of the symbol is motivated by its use in mathematics for describing relationships as "equivalent to" or "identical to". The shape is linked to two ports, that are small arcs attached to the centres of opposite sides of the shape, as shown in Figure 2.52 on the following page.

**Label:**

None.

**Auxiliary items:**

None.

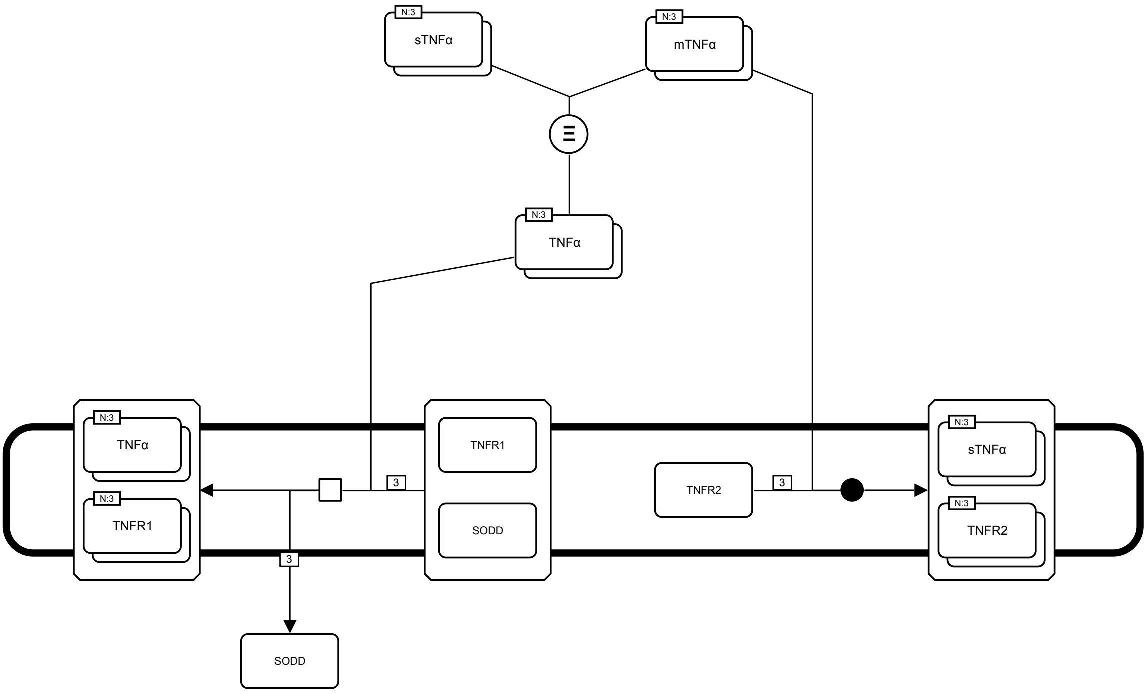

**Figure 2.53:** *Binding of diverse forms of TNFα to TNF receptors.*

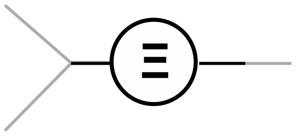

**Figure 2.52:** *The Process Description glyph for* equivalence operator. *Only two inputs are represented, but more would be allowed.*

The example of Figure 2.53 presents the binding of different forms of TNF$\alpha$ to two receptors, TNFR1 and TNFR2. Only membrane TNF$\alpha$ (mTNF$\alpha$) can bind to TNFR2, while both membrane TNF$\alpha$ and soluble TNF$\alpha$ (sTNF$\alpha$) can bind to TNFR1. Binding of both forms to TNFR1 can conveniently be represented using a generic form of TNF$\alpha$, that is built using an *equivalence operator.*

### 2.10 Logic arc

#### 2.10.1 Glyph: *Logic arc*

*Logic arc* is used to represent the fact that an entity pool or a logical operator influences the outcome of a logic operator.

**SBO Term:**

SBO:0000398 ! logical relationship

**Origin:**

One *EPN* (Section 2.4) or *logical operator* (Section 2.9).

**Target:**

One *logical operator* (Section 2.9) or *EPN* (Section 2.4).

**Symbol:**

No particular symbol is used to represent a *logic arc*, as shown in Figure 2.54.

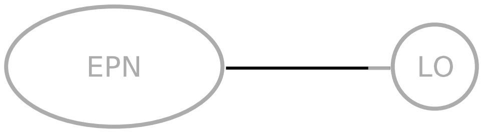

**Figure 2.54:** *The Process Description glyph for* logic arc.

### 2.11 Annotating nodes and arcs

#### 2.11.1 Glyph: *Annotation*

In SBGN Process Description Level 1 there are cases where the language does not capture everything the author wishes to convey. This may be additional experimental detail or descriptions of mechanisms that cannot be fully described by the Process Description language. In this case the language provides the *annotation* glyph. This contains text and is associated with a particular glyph in a map. Importantly, it is purely "decoration" and does alter the meaning the map.

**SBO Term:**
SBO:0000550 ! annotation

**Incoming arcs:**
None.

**Outgoing arcs:**
None.

**Container:**
An *annotation* is represented by a rectangular shape with a folded corner, as shown in Figure 2.55. This shape is linked to the annotated element via a callout (see Figure 2.56). The callout should overlap with the object it is annotating.

**Label:**
An *annotation* is identified by a label that is a string of characters that may be distributed on several lines to improve readability. The centre of the label must be placed on the centre of the shape.

**Auxiliary items:**
None.

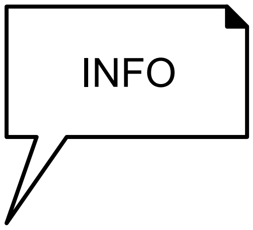

**Figure 2.55:** *The Process Description glyph for* annotation.

### 2.12 Referring to other nodes

Reference nodes handle links or relationships between elements of a map and sub-map. At present there is only one reference glyph, *tag*, which can be used in a map referred to by a *submap* (Section 2.13.1).

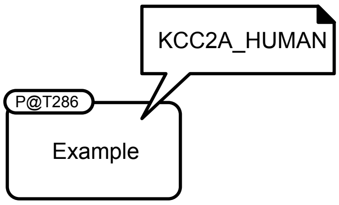

**Figure 2.56:** *Example of* annotations *adding information to the description of the trans-phosphorylation of CaMKII. Note that three different types of links are used between annotation nodes and annotated elements. However, it is recommended to use a consistent scheme within a map.*

#### 2.12.1 Glyph: *Tag*

A *tag* is a named handle, or reference, to another *EPN* (Section 2.4) or *compartment* (Section 2.5.1) of the map. Together with the *submap terminal* (Section 2.3.5), it allows linking glyphs of a map to their counterpart in a submap.

**SBO Term:**
Not applicable.

**Incoming arcs:**
One *equivalence arc* (Section 2.12.2).

**Outgoing arcs:**
None.

**Container:**
A *tag* is represented by a rectangular shape fused to an empty arrowhead, as shown in Figure 2.57. The incoming *equivalence arc* (Section 2.12.2) should be linked to the extremity of the arrowhead.

**Label:**
A *tag* is identified by a label that is a string of characters that may be distributed on several lines to improve readability. The centre of the label must be placed on the centre of the shape. The label may extend outside of the shape.

**Auxiliary items:**
None.

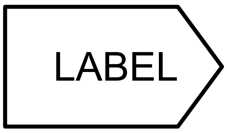

**Figure 2.57:** *The Process Description glyph for* tag*.*

#### 2.12.2 Glyph: *Equivalence arc*

An *equivalence arc* is used to link a *tag* (Section 2.12.1) or a *submap terminal* (Section 2.3.5) to the *EPN* (Section 2.4) or *compartment* (Section 2.5.1) it refers to.

**SBO Term:**
Not applicable.

**Origin:**
One *EPN* (Section 2.4) or *compartment* (Section 2.5.1).

**Target:**
One *tag* (Section 2.12.1) or *submap terminal* (Section 2.3.5).

**Symbol:**
No particular symbol is used to represent an *equivalence arc*, as shown in Figure 2.58.

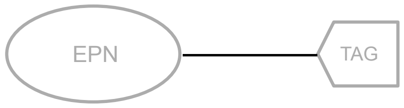

**Figure 2.58:** *The Process Description glyph for* Equivalence arc.

### 2.13 Encapsulation

#### 2.13.1 *Submap*

A *submap* is used to encapsulate a map (including all types of nodes and edges) within one glyph. As such, it is not equivalent to an *omitted process* (Section 2.6.2). The *submap* hides the content of this map to the users, and displays only *submap terminals* (Section 2.3.5). In the case of an SBGN description that is made available through a software tool, the map enclosed by the *submap* may be available to the tool. A user could then ask the tool to expand this map in a different canvas, for instance by clicking on the *submap*. In the case of an SBGN description made available in a book or a website, the content of the map may be available on another page, possibly accessible via an hyperlink on the *submap*.

**SBO Term:**
SBO:0000395 ! encapsulating process

**Incoming arcs:**
None.

**Outgoing arcs:**
None.

**Container:**
A *submap* is represented by a rectangular shape, to remind that it is fundamentally a process.

**Label:**
A *submap* is identified by a label that is an unbordered box containing a string of characters. The characters may be distributed on several lines to improve readability. The centre of the label must be placed on the centre of the shape. The label may extend outside of the shape.

**Auxiliary items:**
A *submap* must carry one or more *submap terminals* (Section 2.3.5), each linked to an *EPN* (Section 2.4) or *compartment* (Section 2.5.1) of the map using an *equivalence arc* (Section 2.12.2).

Figure 2.60 represents a *submap* that encapsulates processes transforming glucose into fructose-6-phosphate. The *submap* carries five *submap terminals*, four linked to *EPNs* and

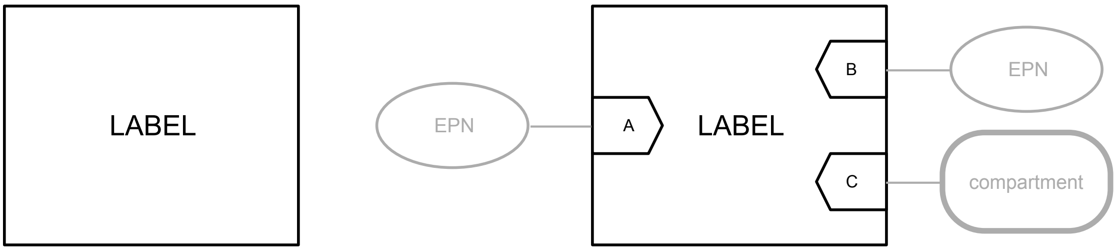

**Figure 2.59:** *The Process Description glyph for* submap, *shown plain and unadorned on the left, and with three* submap terminals *on the right.*

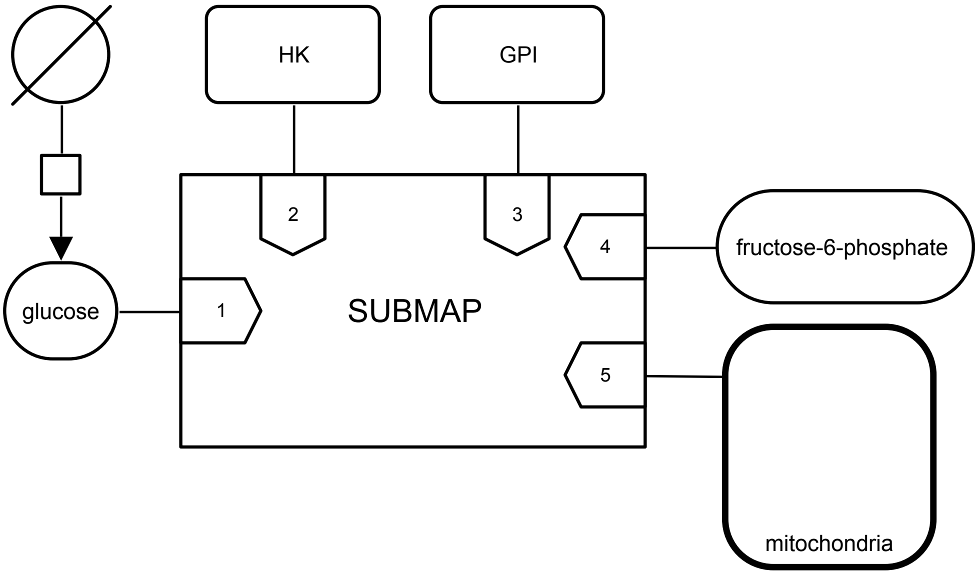

**Figure 2.60:** *Example of a* submap *encapsulating processes that transform glucose into fructose-6-phosphate. The map enclosed by the* submap *is shown in Figure 2.61 on the next page, whose* tags *are referred to by the* submap terminals *decorating the present* submap *glyph.*

one linked to a *compartment*. The latter is particularly important in the case of *EPNs* present only in a *compartment* enclosed in a *submap*, and that are not linked to *submap terminals* themselves. Note that the *submap terminals* do not allow defining a "direction" for the flux of the processes enclosed in the *submap*, which is solely determined by the context as in Figure 2.60.

The map in Figure 2.61 on the following page represents the map enclosed in the *submap* of Figure 2.60. Note that the tag 5 links the mitochondria *compartment* in this map to the mitochondria *compartment* of the main map in Figure 2.61 on the following page. The *compartment* containing glucose-6-phosphate is implicitly defined as the same as the *compartment* containing glucose and fructose-6-phosphate. There is no ambiguity because if glucose and fructose-6-phosphate were in different *compartments*, at least one of them would have been linked to a *submap terminal* of the *submap* of Figure 2.60.

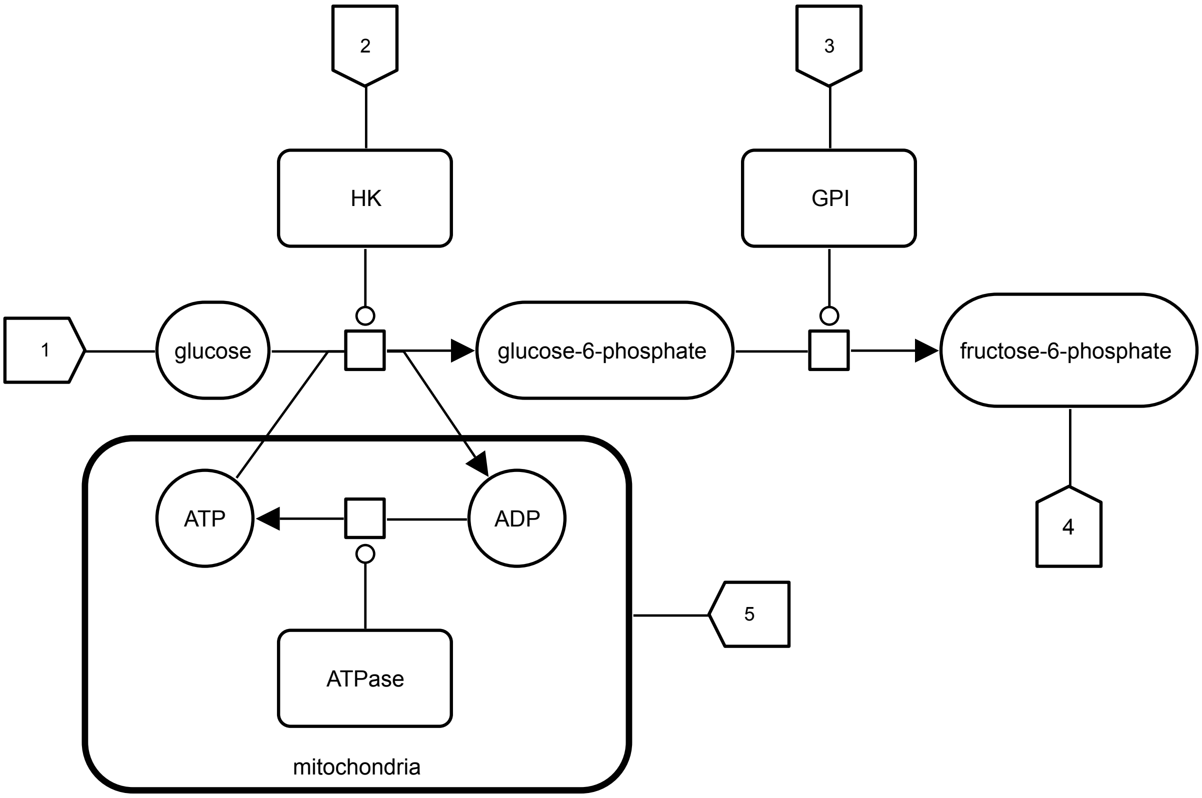

**Figure 2.61:** *Example of a map with* tags, *showing that it is enclosed in a* submap *of another map (here, the one of Figure 2.60 on the previous page).*

## Chapter 3

## Process Description Language Grammar

### 3.1 Overview

One of the goals of SBGN Process Description Level 1 is to provide a visual description of biological systems that can accurately and unambiguously convey to the reader what the writer of a Process Description map meant. A secondary, but nevertheless important goal is to provide a visual language that can be supported by software tools. To achieve both goals we require a detailed set of rules that are universally applied to Process Description maps. In Chapter 2 the glyphs of the Process Description language were introduced and key usage rules were described; we expect that this will be sufficient for casual users of the notation. However, this chapter provides a more detailed description of the rules that apply to the Process Description language and it is anticipated that this will be required reading for advanced users of the notation and tool developers implementing SBGN Process Description viewers or editors.

In addition to understanding the rules of the notation it is also important to understand the underlying model, or abstraction, of the biological world that the Process Description language uses. By understanding the abstractions used one can also understand more clearly what the author of a map meant, and also what the author could not say given the limitations of the notation. In the next section, we describe the abstractions and concepts implicit in the SBGN Process Description language.

### 3.2 Concepts

The key abstraction of the SBGN Process Description language is one of processes that act on pools of entities. The entities are typically biological molecules, but need not be (such as a *perturbing agent*), and the *process* is typically a combination of one or more biochemical reactions, but again need not be. Processes are controlled, or *modulated*, by other entity pools and new entities can be obtained from an *empty set* and existing entities discarded to an *empty set*.

It can be helpful to think of the SBGN Process Description as describing the flow of a fluid, especially when trying to understand concepts such as stoichiometry, and modulation. In this analogy, each *EPN* represents a tank of fluid, which may be emptied via a pipe (the consumption arc, Section 2.7.1) that is connected to a valve (the process node, Section 2.6), which in turn is connected to other pipes (the production arcs, Section 2.7.2) that fill other tanks (*EPNs*). The opening of the valve, and thus the rate of the process can be controlled by the volume of fluid in another tank (this is modulation)[1]. If there are two consumption pipes feeding fluid into the process and one is wider and allows double the flow of the first then the fluid will mix in the process with a 2:1 ratio. This corresponds to the stoichiometry of the *consumption* and *production* arcs. Finally, the system needs a source of fluid from one or more external sources

[1] The precise nature of the relationship between valve opening and amount of fluid determines the nature of the modulation.

(represented with the *empty set*, see section Section 2.4.7) and one or more sinks for it to drain away (also represented with the *empty set*).

One can see that this maps very closely to the abstraction described in the Process Description language, but it also has an interesting side-effect that it allows us to add quantities to SBGN glyphs. In particular, EPNs have an implicit amount of entities in the entity pool and processes have an implicit rate. Since SBGN Process Description is not a modelling language this will not be discussed in any further detail, however, this is an important concept that underpins our understanding of how processes are modulated in Section 3.5.2.4.

### 3.3 The conceptual model

In order to formalize the conceptual representation described above and the semantic rules that we will describe later, we have described the Process Description language using UML Domain Object Model. We have used it to define the "taxonomy" of the Process Description glyphs and their relationship to each other. Finally we describe the attributes of each node glyph that are required to uniquely identify an instance of it in a Process Description map. The concept of identity is important in some of the rules described below.

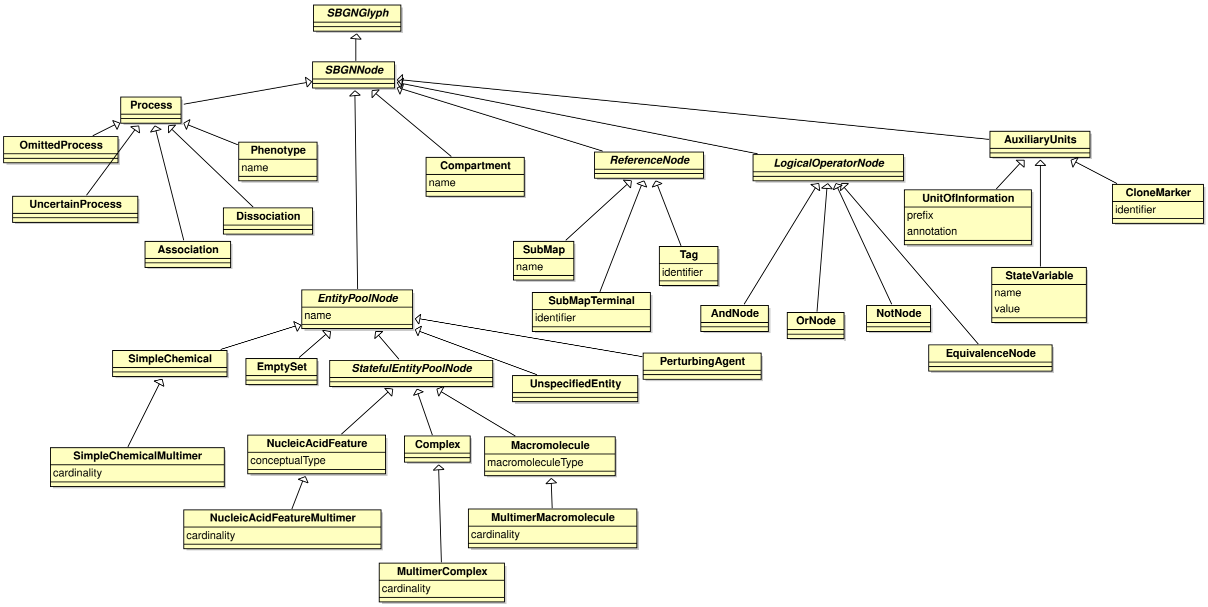

**Figure 3.1:** *Organisation of the node glyphs within SBGN Process Description language. All UML classes (boxes) correspond to Process Description node glyphs except those with italicised names, which are organisational groupings. They correspond to the groupings used elsewhere in this document.*

**Table 3.1:** *The Identifying attributes of Process Description node glyphs. When a glyph is always unique in a map, this is indicated by the term* instance*. The term* state values *indicates that the values of all the EPN's state variables are used in the definition of its identity.*

| Glyph | Identifying Attributes |
|---|---|
| Unit of Information | *instance* |
| State variable | Owning StatefulEpnNode, value |

*continued on next page*

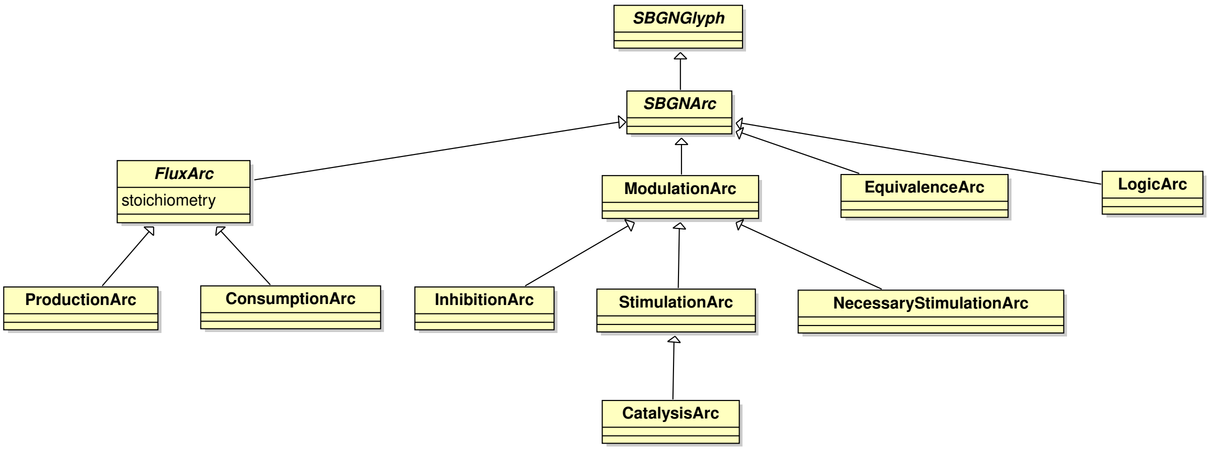

**Figure 3.2:** *Organisation of the edge glyphs within SBGN Process Description language. All UML classes (boxes) correspond to Process Description node glyphs except those with italicised names, which are organisational groupings. They correspond to the groupings used elsewhere in this document.*

*continued from previous page*

| **Glyph** | **Identifying Attributes** |
|---|---|
| Clone maker | Owning EpnNode |
| Labelled clone maker | Owning EpnNode, Label |
| Unspecified entity | Owning compartment, Label |
| Simple chemical | Owning compartment, Label |
| Macromolecule | Owning compartment, Label, Material type, *state values* |
| Nucleic acid feature | Owning compartment, Label, Conceptual type, *state values* |
| Complex | Owning compartment, Label, *state values* |
| Simple chemical multimer | Owning compartment, Label, Cardinality |
| Macromolecule multimer | Owning compartment, Label, Material type, Cardinality, *state values* |
| Nucleic acid feature multimer | Owning compartment, Label, Conceptual type, Cardinality, *state values* |
| Complex multimer | Owning compartment, Label, Cardinality, *state values* |
| Empty set | *instance* |
| Perturbing agent | Label |
| Tag | Label |
| Submap terminal | Label |
| Compartment | Label |
| Submap | Label |
| Process | *instance* |
| Omitted process | *instance* |
| Uncertain process | *instance* |
| Association | *instance* |
| Dissociation | *instance* |
| Phenotype | *instance* |

*Notes*

A complex may have a Label or be defined by its subunits. In the case where it has both then all complexes with the same label must also have the same subunit composition.

### 3.4 Syntax

The syntax of the SBGN Process Description language is defined in the form of an incidence matrix. An incidence matrix has arcs as rows and nodes as columns. Each element of the matrix represents the role of an arc in connection to a node. Source (S) means that the arc can begin at that node. Target (T) indicates that the arc can end at that node. Numbers in parenthesis represent the maximum number of arcs of a particular type to have this specific connection role with the node. Empty cells means the arc is not able to connect to the node.

#### 3.4.1 Node connectivity

| *Arc\EPN* | *macromolecule* | *simple chemical* | *unspecified entity* | *multimer* | *complex* | *nucleic acid feature* | *tag* | *submap terminal* | *empty set* | *perturbing agent* | *submap* |
|---|---|---|---|---|---|---|---|---|---|---|---|
| *consumption* | S | S | S | S | S | S | | | S(1) | | |
| *production* | T | T | T | T | T | T | | | T(1) | | |
| *modulation* | S | S | S | S | S | S | | | | S | |
| *stimulation* | S | S | S | S | S | S | | | | S | |
| *catalysis* | S | S | S | S | S | | | | | | |
| *inhibition* | S | S | S | S | S | S | | | | S | |
| *necessary stimulation* | S | S | S | S | S | S | | | | S | |
| *logic arc* | S | S | S | S | S | S | | | | | |
| *equivalence arc* | S | S | S | S | S | S | T | T | | | |

*Additional rules*

1. An EPN that is a *subunit* of a *complex* can only be linked to a modulation arc.
2. With the above exception, glyphs that are subunits cannot be connect to any arc glyph.
3. A *logic arc* that is linked to an *equivalence operator* on one side can only be linked to an EPN on the other side.

| *Arc\PN* | *process* | *omitted process* | *uncertain process* | *phenotype* | *association* | *dissociation* | *and* | *or* | *not* | *equivalence* |
|---|---|---|---|---|---|---|---|---|---|---|
| *consumption* | T | T | T | | T | T(1) | | | | |
| *production* | S | S | S | | S(1) | S | | | | |
| *modulation* | T | T | T | T | | | S(1) | S(1) | S(1) | |
| *stimulation* | T | T | T | T | | | S(1) | S(1) | S(1) | |
| *catalysis* | T | T | T | T | | | S(1) | S(1) | S(1) | |
| *inhibition* | T | T | T | T | | | S(1) | S(1) | S(1) | |
| *necessary stimulation* | T | T | T | T | | | S(1) | S(1) | S(1) | |
| *logic arc* | | | | | | | T | T | T(1) | T |
| *equivalence arc* | | | | | | | | | | |

#### 3.4.2 Containment definition

By containment we mean that a glyph can be drawn inside the other glyph. This does not necessarily mean that the glyph "belongs" the the containing node, although in some cases it does. In this section the concept of "belonging" is referred to as ownership and you can find node ownership in Section 3.3. There are two glyphs that allow containment: *compartment* and *complex*. The next table describes the relationship between Process Description glyphs and these containers. A + means that the element may be drawn within a container. A − means containment is not allowed.

| Glyph \ Containers | *complex* | *compartment* |
|---|---|---|
| *unspecified entity* | + | + |
| *simple chemical* | + | + |
| *macromolecule* | + | + |
| *nucleic acid feature* | + | + |
| *multimer* | + | + |
| *empty set* | - | + |
| *perturbing agent* | - | + |
| *phenotype* | - | + |
| *tag* | - | + |
| *submap terminal* | - | + |
| *complex* | + | + |
| *compartment* | - | + |
| *submap* | - | + |
| *process* | - | + |
| *omitted process* | - | + |
| *uncertain process* | - | + |
| *association* | - | + |
| *dissociation* | - | + |
| *consumption* | - | + |
| *production* | - | + |
| *modulation* | - | + |
| *stimulation* | - | + |
| *catalysis* | - | + |
| *inhibition* | - | + |
| *necessary stimulation* | - | + |
| *logic arc* | - | + |
| *equivalence arc* | - | + |
| *and* | - | + |
| *or* | - | + |
| *not* | - | + |
| *equivalence* | - | + |

### 3.5 Semantic rules

#### 3.5.1 EPNs

1. All *state variables* associated with a Stateful Entity Pool Node should be unique and not duplicated within that node.

2. If a state variable is used in one EPN then is must be used in all equivalent stateful EPNs[2].

3. EPNs should not be orphaned (i.e. they must be associated with at least one arc).

[2]A stateful EPN is equivalent if the EPNs are identical when their state descriptions are ignored.

#### 3.5.2 Process Nodes

As described in Section 2.6.1, the *consumption* and *production* arcs converge before connecting to the process node (Figure 3.3). This defines the EPNs that are the input and outputs of an irreversible process. Since, processes can be reversible in the following rules we refer to these groupings as the "left-hand-side" (LHS) and "right-hand-side" (RHS) of the process[3]. For convenience we will also collectively refer to the *consumption* and *production* arcs as *flux* arcs.

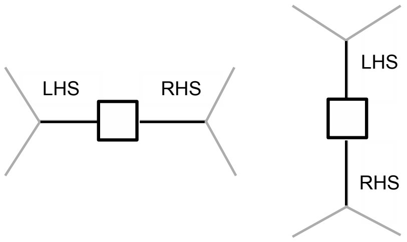

**Figure 3.3:** *An illustration of the "sidedness" of a process. The designation of LHS and RHS is essentially arbitrary.*

##### 3.5.2.1 Flux Arcs

1. All process nodes (with the exception of *phenotype*) must have a LHS and RHS.
2. All EPNs on the LHS of a process must be unique.
3. All EPNs on the RHS of a process must be unique.
4. All *phenotype* glyphs must be associated with at least one modulation arc.
5. The EPNs that make up the LHS of the process should be consistent with the RHS, i.e., the process should constitute a balanced biochemical reaction.
6. Once the stoichiometry of a flux arc is displayed in a map then all other flux arcs should display their stoichiometry make.
7. If the stoichiometry is undefined or unknown this should be indicated by the use of a question mark ("?").
8. If more than one set of stoichiometries can be applied to the flux arcs of the process then the stoichiometry of the flux arcs must be displayed.

##### 3.5.2.2 Association

1. An *Association* is always an irreversible process.
2. If a *Complex* is on the RHS of the association then there must be at least 2 EPNS on the LHS.

##### 3.5.2.3 Dissociation

1. A *Dissociation* is always an irreversible process.
2. If a *Complex* is on the LHS of the dissociation then there must be at least 2 EPNs on the RHS.

[3]Note this designation is purely for grouping and is used even then the sides of the reaction are above and below the process.

##### 3.5.2.4 Modulation

As discussed in Section 3.2, it is implied, but not defined explicitly that the process has a rate at which it converts its LHS EPNs to its RHS EPNs (and vice-versa in the case of a reversible process). This concept is important in understanding how the Process Description language describes process modulation.

1. A *process* with no modulations has an underlying "basal rate" which describes the rate at which it converts inputs to outputs.
2. A *modulation* changes the basal rate in an unspecified fashion.
3. A *stimulation* is a modulation that increases the basal rate.
4. An *inhibition* is a modulation that decreases the basal rate.
5. The above types of modulation, when assigned to the same process, are combined and have a multiplicative effect on the basal rate of the process.
6. Modulators that do not interact with each other in the above manner, should be drawn as modulating different process nodes. Their effect is therefore additive.
7. At most one *necessary stimulation* can be assigned to a process node. Two *necessary stimulations* would imply an implicit AND or OR operator. For clarity only one *necessary stimulation* can be assigned to a *process*, and such combinations must be explicitly expressed using *logical operators*.
8. At most one *catalysis* can be assigned to a *process*. Modulation by a catalysis arc implies that the exact biochemical mechanism underlying the process is known. In this context two *catalysis* cannot be assigned to the same process node as they represent independent reactions. Other EPNs can be assigned to the same process as a catalysis, such as modulators, stimulators, and inhibitors, and will have a multiplicative modulation on the reaction rate defined by the catalysis.

##### 3.5.2.5 Reversible Processes

A process is deemed to be reversible if it has *production* arcs on both the LHS and RHS of a process node Figure 3.4. Semantically, the *production* arc can be thought of as allowing a reversible flow of entities between the *process* and the *EPN*. A *consumption* arc only permits an irreversible flow from the *EPN* to the *process*. In this way, the *consumption* arc forces the *process* to be irreversible. *Consumption* arcs cannot be associated with both sides of a *process* as this would prohibit any flow through the *process*.

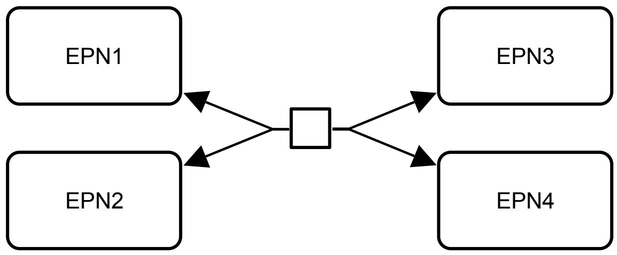

**Figure 3.4:** *A valid reversible process. A process is reversible if its LHS and RHS contain only* production *arcs.*

1. A mixture of *consumption* and *production* arcs on the same side of a *process* is not permitted.

2. The semantics of *modulation* is the same as for irreversible processes, .i.e., the amount of entity in the modulation pool affects the rate of the process.

#### 3.5.3 Cloning

SBGN allows identical nodes to be duplicated on a map if they are explicitly marked as such. This is done using a *clone marker*. The details are shown in table 3.2.

**Table 3.2:** *Duplication rules.*

| Node | Can be duplicated | Indication | Additional Rules |
|---|---|---|---|
| Compartment | N | | |
| SimpleChemical | Y | *Simple clone marker* | |
| UnspecifiedEntity | Y | *Simple clone marker* | |
| EmptySet | Y | | |
| Perturbing Agent | Y | *Simple clone marker* | |
| Phenotype | Y | *Simple clone marker* | |
| MultimerChemicalEntity | Y | *Simple clone marker* | |
| StatefulEntityPool | Y | *Labeled clone marker* | |
| Macromolecule | Y | *Labeled clone marker* | |
| MultimerMacromolecule | Y | *Labeled clone marker* | |
| NucleicAcidFeature | Y | *Labeled clone marker* | |
| Complex | Y | *Labeled clone marker* | |
| Process | Y | None | |
| OmittedProcess | Y | As Process | |
| UncertainProcess | Y | As Process | |
| Association | Y | As Process | |
| Dissociation | Y | As Process | |
| LogicalOperator | Y | None | |
| AND | Y | None | |
| OR | Y | None | |
| NOT | Y | None | |
| Equivalence | Y | None | |

#### 3.5.4 Compartment spanning

An *EPN* cannot *belong* to more than one *compartment*. However, an EPN can be *drawn* over more than one *compartment*. In such cases, the decision on which is the owning *compartment* is deferred to the drawing tool or the author. A *complex* may contain EPNs which belong to different *compartments* and in this way a *complex* can be used to describe entities that span more than one compartment.

This restriction makes it impossible to represent in a semantically correct way a macromolecule that spans more then one compartment — for example a receptor protein. It is clearly desirable to be able to show a macromolecule in a manner that the biologist expects (i.e., spanning from the outside through the membrane to the inside). Therefore, the author is recommended to draw the macromolecule across compartment boundaries, but the underlying SBGN semantic model will assign it to only one. Note that this has implications for auto-layout algorithms as they will only be able to treat such *entity pool nodes* as contained within a *compartment* and will have no way of knowing a macromolecule spans a compartment.

The current solution is consistent with other Systems Biology representations such as SBML and BioPAX. For more information about the problems representing membrane spanning proteins and the rationale behind the current solution see Section D.

#### 3.5.5 Submaps

The submap is a visual device that allows the detail of an Process Description map to be exported into another Process Description map and replaced by a *submap* glyph, which acts as a place-holder. This is described and illustrated in Section 2.13.1. In the following discussion we will refer to the original map as the *main* map and the map containing the export detail as the submap.

1. For a valid mapping between an EPN in the map and submap to exist the identifiers in the *tag* and the submap terminal must be identical and their associated entity pool nodes must be identical.

2. If the same EPN is present in the map and a submap, then they must be mapped to each other.

3. Since the main map and submap share the same namespace, an EPN that is cloned in the main map must also be marked as cloned in the submap — even if there is only one copy of the EPN in the submap. The converse applies when the EPN in the submap is cloned[4].

#### 3.5.6 Equivalence operator

1. Pools should not overlap

2. A *process* should not affect *EPNs* belonging or deriving from both sides of an *equivalence operator*

3. For *simple chemicals* (stateless *EPNs*) it is not allowed to show specifics both as input and as output of a pathway

For automatic verification, the first rule can be transformed into the following validation rules:

(a) Split generic processes and influences into specific ones.

(b) There should not be any repeating processes or influences after this splitting procedure is complete.

[4]This has the additional benefit of ensuring that main maps and submaps do not need to be modified if the submap is exanded and collapsed by a viewing or editing tool.

## Chapter 4

## Layout Rules for a Process Description

### 4.1 Introduction

The previous chapters describe the appearance and meaning of SBGN Process Description Level 1 components. Here we describe rules governing the visual appearence and asthetics of the Process Description language. The components of a Process Description have to be placed in a meaningful way – a random distribution with spaghetti-like connections will most likely hide the information encoded in the underlying model, whereas an elegant placement of the objects, giving a congenial appearance of the maps, may reveal new insights. The arrangement of components in a map is called a *layout*.

SBGN Process Descriptions should be easily recognisable not only by the glyphs used, but also by the general style of the layout. However, the arrangement of the components is a complex art in itself, and there is no simple rule which can be applied to all cases. Therefore this section provides rules for the layout of process description maps, divided into two categories:

1. requirements, i.e., rules which **must** be fulfilled by a layout, and

2. recommendations, i.e., rules which **should** be followed if possible.

In addition, we provide a list of additional suggestions which may help in producing aesthetically more pleasant layouts, possibly easier to understand.

Those layout rules are independent of the method used to produce the map, and apply to both manually drawn maps as well as maps produced by an automatic layout algorithm. The rules do not deal with interactive aspects (e. g. the effect of zooming). Further information about automatic network layout (graph drawing) can be found, for example, in the books of Di Battista and co-authors [2], and Kaufmann and Wagner [3].

Please note that the color of objects do not carry any meaning in SBGN. Although one can use colours to emphasize part of a map or encode additional information, the meaning of the map should not depend on the colours. Furthermore, objects can have different sizes and size is also meaningless in SBGN. For example, a process node may be larger than a protein node. Also the meaning of a graph should be conserved upon scaling as far as possible.

### 4.2 Requirements

Requirements are rules which **must** be fulfilled by a layout to produce a valid Process Description map.

#### 4.2.1 Node-node overlaps

Nodes are only allowed to overlap in two cases when they are allowed to contain other nodes—as described in Section 3.4.2. Otherwise, nodes are not allowed to overlap (Figure 4.1 on the following page). This includes the touching of nodes. Touching is not allowed apart from the case where it has a specific meaning, e.g., two macromolecules touching each other within a complex because they form the complex.

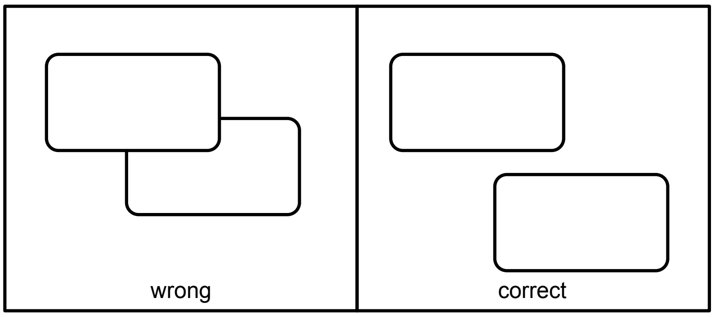

**Figure 4.1:** *Nodes must not overlap.*

#### 4.2.2 Node-edge crossing

Edges must be drawn on the top of a the node (Figure 4.2). See also recommendation Section 4.3.1.

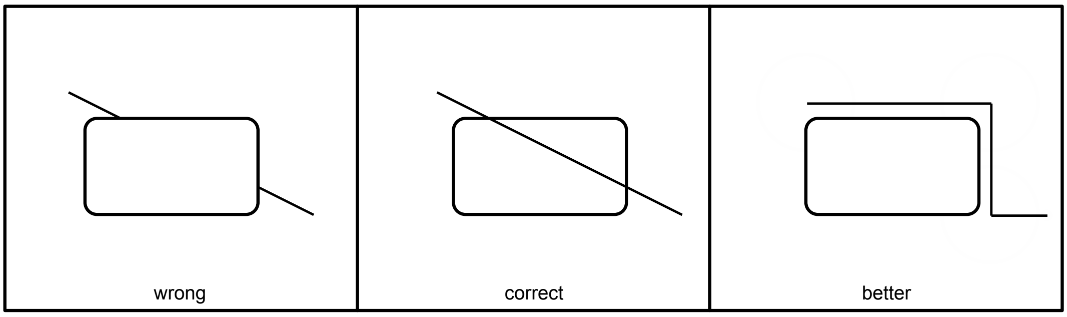

**Figure 4.2:** *If an edge crosses a node, the edge must be drawn on top of the node.*

#### 4.2.3 Node border-edge overlaps

Edges are not allowed to overlap the border lines of nodes (Figure 4.3 on the following page).

#### 4.2.4 Edge-edge overlaps

Edges are not allowed to overlap (Figure 4.4 on the next page). This includes touching of edges. Furthermore, an edge is neither allowed to cross itself nor to cross the boundary of node more than twice or other edges more than once.

#### 4.2.5 Node orientation

Nodes have to be drawn horizontally or vertically, any other rotation of elements is not allowed (Figure 4.5 on the following page).

#### 4.2.6 Node-edge connection

1. The arcs linking the square glyph of a *process* to the *consumption* and *production arcs* are attached to the centre of opposite sides (Figure 4.6 on page 59).

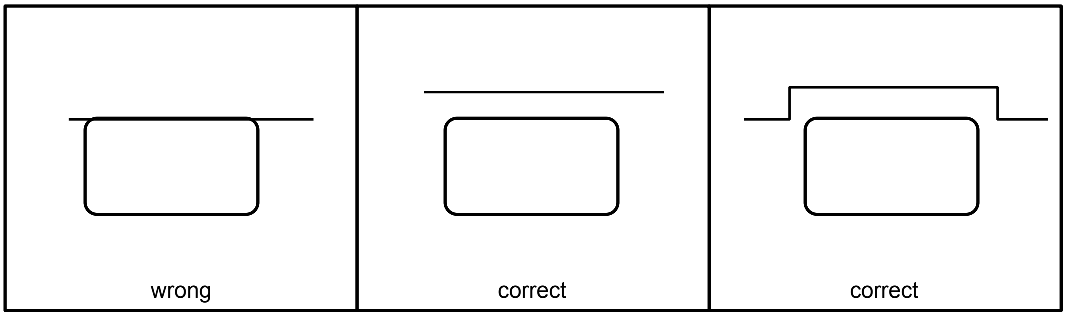

**Figure 4.3:** *Edges must not overlap node borders.*

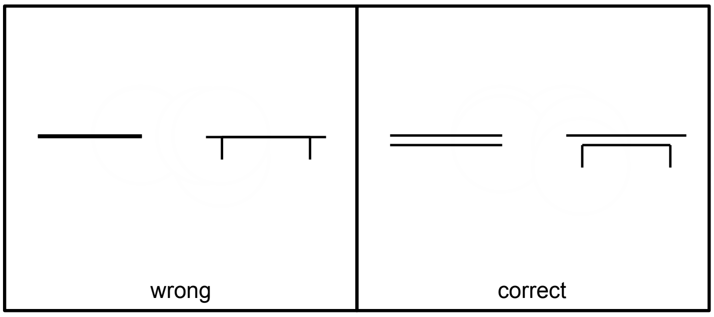

**Figure 4.4:** *Edges must not overlap.*

2. The modulatory arcs are attached to the other two sides, but not necessarily all to the centre, as several modifiers can affect the same process node.

#### 4.2.7 Node labels

At least a part of the label (unbordered box containing a string of characters) has to be placed inside the node it belongs to. Node labels are not allowed to overlap other nodes or other labels (this includes touching of other nodes or labels).

#### 4.2.8 Edge labels

Edge labels are not allowed to overlap nodes. This includes touching of nodes.

#### 4.2.9 Compartments

If a process has all participants in the same compartment the process node and all edges/arcs should be drawn in this compartment. If a process has participants in at least two different compartments, the process node has to be either in a compartment where the process has at least one participant or in the empty space.

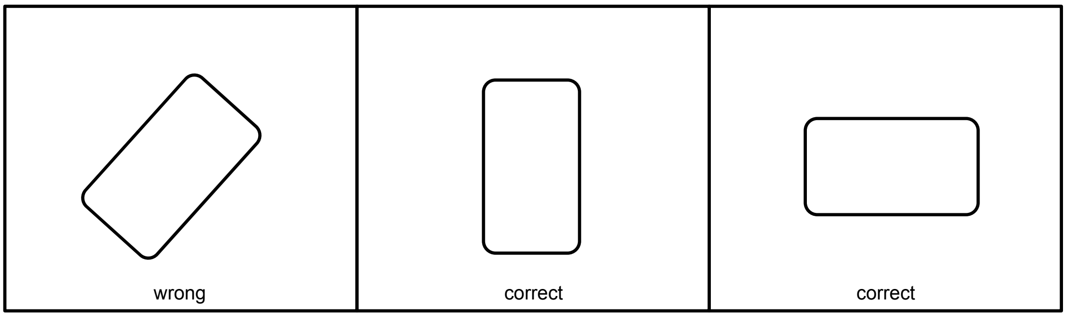

**Figure 4.5:** *The node orientation must be horizontally or vertically.*

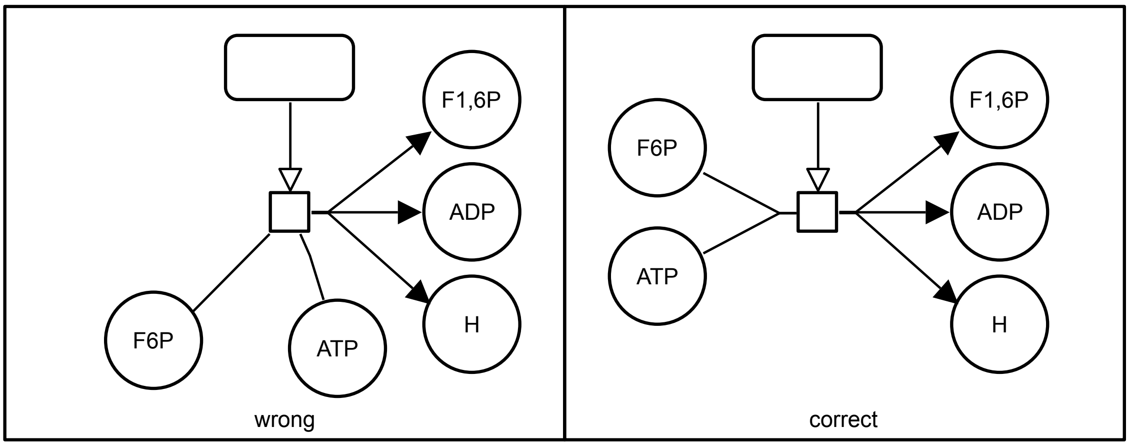

**Figure 4.6:** *Arcs between a* process *and the* consumption *and* production arcs *must be attached to the centre of opposite sides, modulatory arcs must be attached to the other two sides.*

### 4.3 Recommendations

Recommendations are rules which should be followed if possible and generally should improve the clarity of the diagram.

#### 4.3.1 Node-edge crossing

Situations where edges and nodes cross should be avoided. Note that some crossings may be unavoidable, e.g., the crossing between an edge and a compartment border or an edge and a complex (if the edge connects an element inside the complex with something outside).

#### 4.3.2 Labels

Labels should be horizontal. Node labels should be placed completely inside the node if possible. Edge labels should be placed close to the edge and avoid overlapping the edge as well as other edge labels.

#### 4.3.3 Avoid edge crossings

The amount of crossings between edges should be minimized.

#### 4.3.4 Branching of *association* and *dissociation*

The branching points of *association* and *dissociation* nodes should be placed close to the symbol of the process, if possible at a distance comparable to, or smaller than, the diameter of the symbol defining the process (Figure 4.7 on the following page).

#### 4.3.5 Units of information

Units of information should not hide the structure of the corresponding node and should not overlap other elements (Figure 4.8 on the next page).

### 4.4 Additional suggestions

Here is a list of additional layout suggestions which may help improve the asthetics and clarity of Process Description maps.

- Angle of edge crossings: If edge crossing cannot be avoided then the edges should cross with an angle close to 90°.

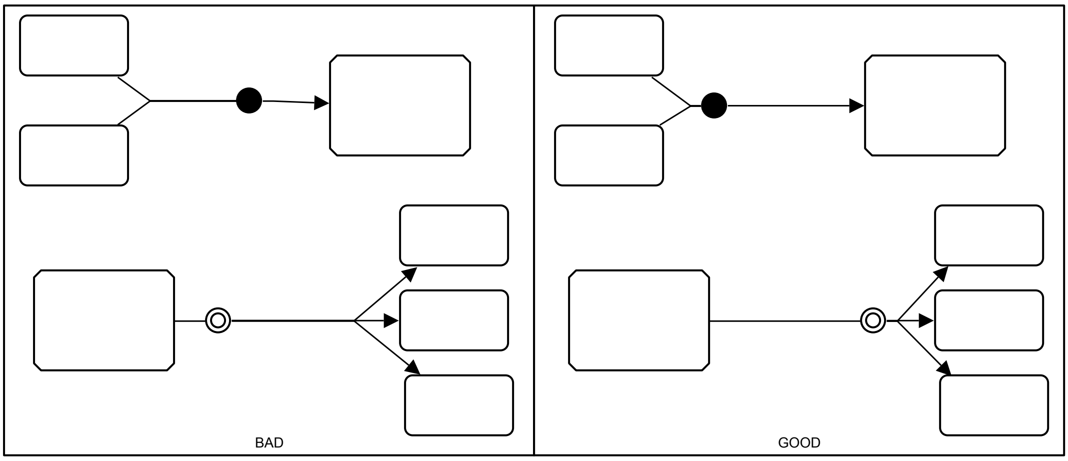

**Figure 4.7:** *Branching points should be close to association and dissociation symbols.*

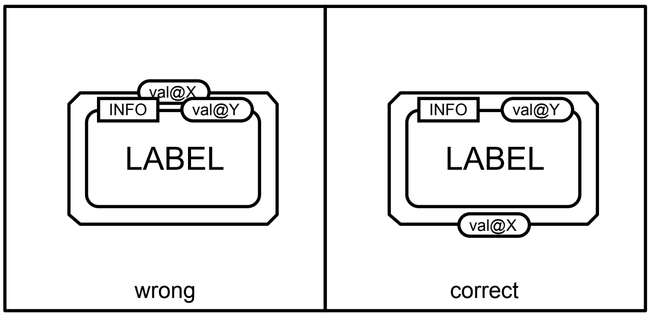

**Figure 4.8:** *Units of information should not overlap with any other element.*

- Drawing area and width/height ratio: The drawing should be compact and the ratio between the width and the height of the drawing should be close to 1.
- Edge length: Long edges should be avoided.
- Number of edge bends: Edges should be drawn with as few bends as possible.
- Similar and symmetric parts: Similar parts of a map should be drawn in a similar way, and symmetric parts should be drawn symmetrically.
- Proximity information: Related elements (e.g., nodes connected by a process or all elements within a compartment) should be drawn close together.
- Directional information: Subsequent processes (e.g., a sequence of reactions) should be drawn in one direction (e.g., from top to bottom or from left to right).
- Compartments: It can help clarity to use a different background shade or color for each compartment.

## Chapter 5

## Acknowledgements

Here we acknowledge those people and organisations that assisted in the development of this and previous releases of the SBGN Process Description language specification. First we specifically acknowledge those who contributed directly to each revision of the specification document, followed by a comprehensive acknowledgement of contributors that attended workshops and forum meetings or in some other way provided input to the standard. Finally, we acknowledge the bodies that provided financial support for the development of the standard.

### 5.1 Level 1 Release 1.0

The specification of was written by Nicolas Le Novère, Stuart Moodie, Anatoly Sorokin, Michael Hucka, Falk Schreiber, Emek Demir, Huaiyu Mi, Yukiko Matsuoka, Katja Wegner, and Hiroaki Kitano. In addition, the specification benefited much from the help of (in alphabetical order) Frank Bergmann, Sarala Dissanayake, Ralph Gauges, Peter Ghazal, Lu Li, and Steven Watterson.

### 5.2 Level 1 Release 1.1

The specification of SBGN PD L1 V1.1 was written by Stuart Moodie and Nicolas Le Novère, with contributions from (in alphabetical order) Frank Bergmann, Sarah Boyd, Emek Demir, Sarala Wimalaratne, Yukiko Matsuoka, Huaiyu Mi, Falk Schreiber, Anatoly Sorokin, and Alice Villéger.

### 5.3 Level 1 Release 1.2

The specification of SBGN PD L1 V1.2 was modified by Stuart Moodie, with contributions from (in alphabetical order) Sarah Boyd, Nicolas Le Novère, and Huaiyu Mi.

### 5.4 Level 1 Release 1.3

The specification of SBGN PD L1 V1.3 was modified by Stuart Moodie, with contributions from (in alphabetical order) Tobias Czauderna, Nicolas Le Novère, and Anatoly Sorokin.

### 5.5 Level 1 Release 2.0

The specification of SBGN PD L1 V2.0 was modified by Adrien Rougny, with contributions from (in alphabetical order) Irina Balaur, Michael Blinov, Hanna Borlinghaus, Ugur Dogrusoz, Andreas Dräger, Augustin Luna, Alexander Mazein, Stuart Moodie, and Vasundra Touré.

### 5.6 Comprehensive list of acknowledgements

Here is a more comprehensive list of people who have been actively involved in SBGN development, either by their help designing the languages, their comments on the specification, help with development infrastructure or any other useful input. We intend this list to be complete. We are very sorry if we forgot someone, and would be grateful if you could notify us of any omission.

Mirit Aladjemm, Irina Balaur, Frank Bergmann, Michael Blinov, Hanna Borlinghaus, Sarah Boyd, Laurence Calzone, Melanie Courtot, Emek Demir, Ugur Dogrusoz, Andreas Dräger, Tom Freeman, Akira Funahashi, Ralph Gauges, Peter Ghazal, Samik Ghosh, Igor Goryanin, Michael Hucka, Akiya Jouraku, Hideya Kawaji, Douglas Kell, Sohyoung Kim, Hiroaki Kitano, Kurt Kohn, Fedor Kolpakov, Nicolas Le Novère, Lu Li, Augustin Luna, Yukiko Matsuoka, Alexander Mazein, Huaiyu Mi, Stuart Moodie, Adrien Rougny, Sven Sahle, Chris Sander, Herbert Sauro, Esther Schmidt, Falk Schreiber, Jacky Snoep, Anatoly Sorokin, Jessica Stephens, Linda Taddeo, Vasundra Touré, Steven Watterson, Alice Villéger, Katja Wegner, Sarala Wimalaratne, Guanming Wu.

The authors are also grateful to all the attendees of the SBGN meetings, as well as to the subscribers of the `sbgn-discuss@googlegroups.com` mailing list.

### 5.7 Financial Support

The development of SBGN was mainly supported by a grant from the Japanese *New Energy and Industrial Technology Development Organization* (NEDO, `http://www.nedo.go.jp/`). The *Okinawa Institute of Science and Technology* (OIST, `http://www.oist.jp/`), the *AIST Computational Biology Research Center* (AIST CBRC, `http://www.cbrc.jp/index.eng.html`) the British *Biotechnology and Biological Sciences Research Council* (BBSRC, `http://www.bbsrc.ac.uk/`) through a Japan Partnering Award, the European Media Laboratory (EML Research gGmbH, `http://www.eml-r.org/`), and the Beckman Institute at the California Institute of Technology (`http://bnmc.caltech.edu`) provided additional support for SBGN workshops. Some help was supplied by the *Japan Science and Technology Agency* (JST, `http://www.jst.go.jp/`) and the *Genome Network Project* of the Japanese Ministry of Education, Sports, Culture, Science, and Technology (MEXT, `http://www.mext.go.jp/`) for the development of the gene regulation network aspect of SBGN, and from the *Engineering and Physical Sciences Research Council* (EPSRC, `http://www.epsrc.ac.uk`) during the redaction of the specification. The *German Ministry of Education and Research* (`https://www.bmbf.de/`) provided support for an SBGN workshop and the redaction of the specification. Funding from *National Institute of General Medical Sciences (NIGMS)* grant (P41 GM103504) and *National Human Genome Research Institute (NHGRI)* grant (U41 HG006623) was additionally used for the redaction of the specification.

## Appendix A

## Complete examples of Process Description Maps

The following maps present complete examples of SBGN Process Descriptions representing biological processes. They by no mean exhaust the possibilities of SBGN Process Description Level 1.

Figure A.1 presents an example of metabolic pathway, that examplifies the use of the *EPNs simple chemical*, *macromolecule*, and *clone marker*, the *PNs process*, and the *connecting arcs consumption*, *production* and *catalysis.*

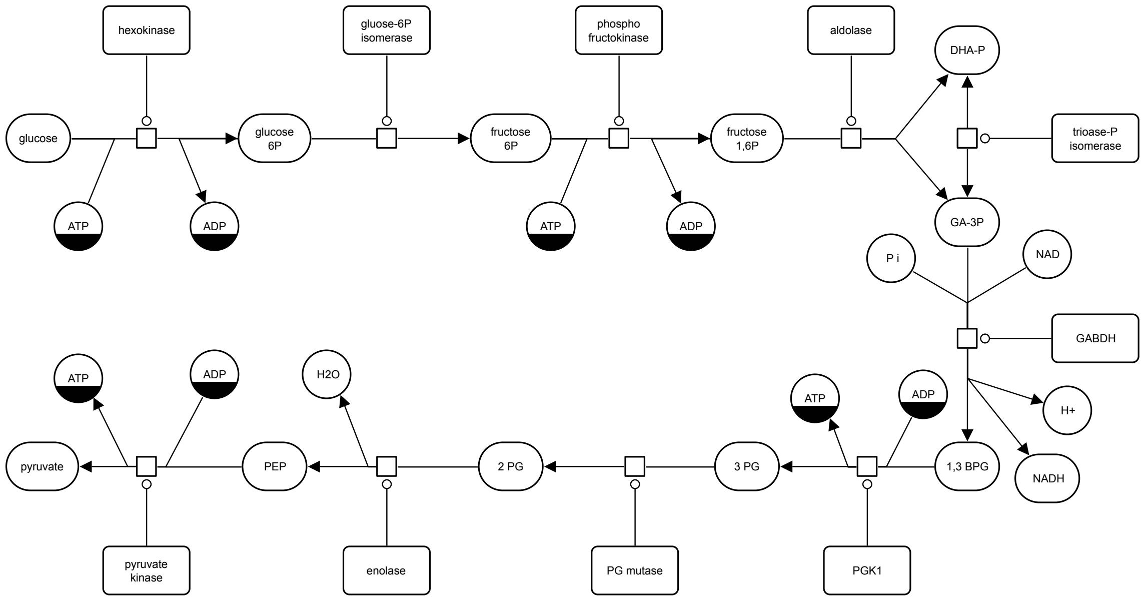

**Figure A.1:** *Glycolysis. This example illustrates how SBGN can be used to describe metabolic pathways.*

Figure A.2 on the next page presents an example of signalling pathway, that examplifies in addition the use of the *EPNs phenotype*, and *state variable*, the *containers complex*, *compartment* and *submap*, the *PNs association*, and the *connecting arcs stimulation.* Note the complex IGF and IGF receptor, located on the boundary of the compartment. This position is only for user convenience. The complex has to belong to a given compartment in SBGN Process Description Level 1.

Figure A.3 on page 65 is an expanded version of the submap present on the map present in

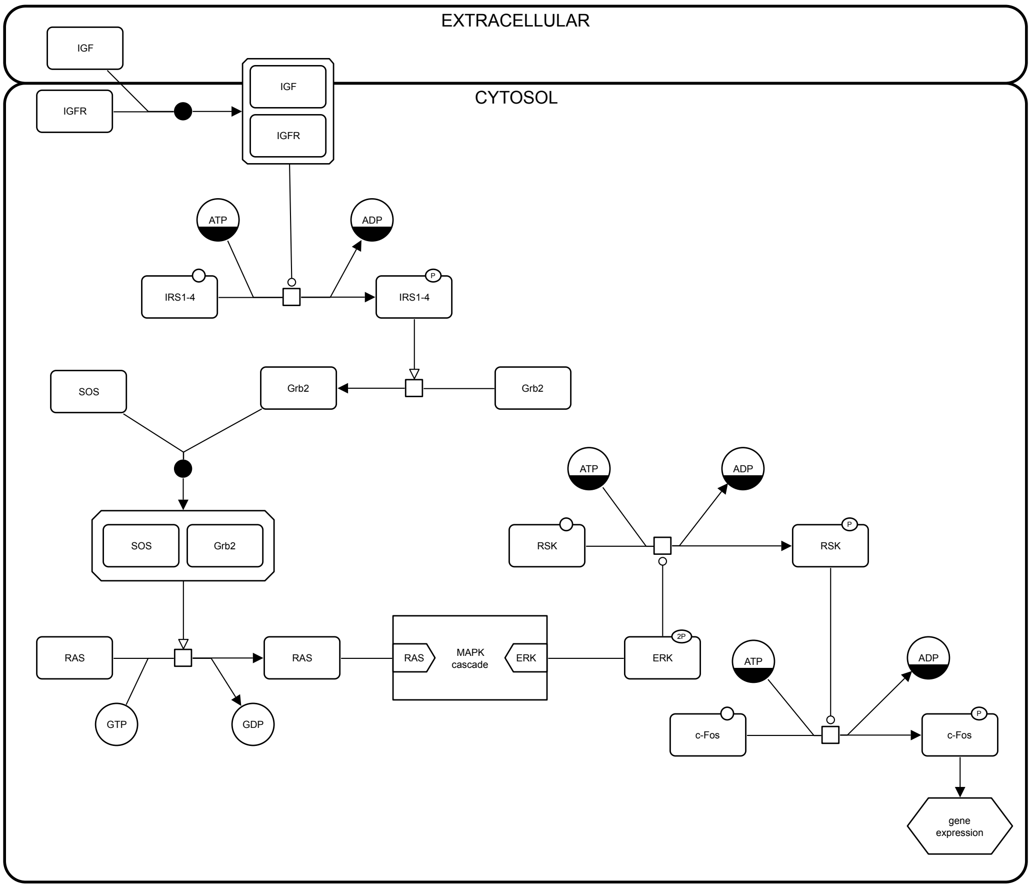

**Figure A.2:** *Insulin-like Growth Factor (IGF) signalling. This example shows the use of compartments and how details can be hidden by using a submap. The submap is shown on Figure A.3 on the next page.*

Figure A.2. It shows the use of *tag*.

Figure A.4 on page 66 introduces an SBGN Process Description that spans several compartments. Note that the compartment "synaptic vesicle" is not **contained** in the compartment "synaptic button" but **overlaps** it. The *simple chemical* "ACh" of the "synaptic vesicle" is not the same *EPN* than the "ACh" of the "synaptic button" and of "synaptic cleft". The situation is similar with the compartments "ER" and "muscle cytosol". The map exemplifies the use of the *PN omitted* and *dissociation*, and the *connecting arc necessary stimulation.*

Figure A.5 on page 67 introduces the use of SBGN Process Description Level 1 to encode gene-regulatory networks. It also show the use of the *EPNs empty set* and the *logical operator and.*

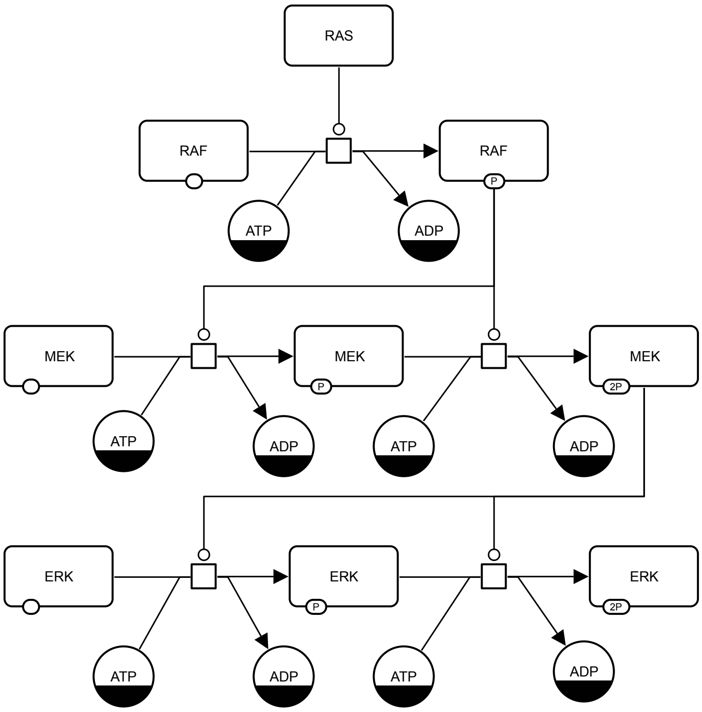

**Figure A.3:** *A submap of the previous map showing the MAPK cascade.*

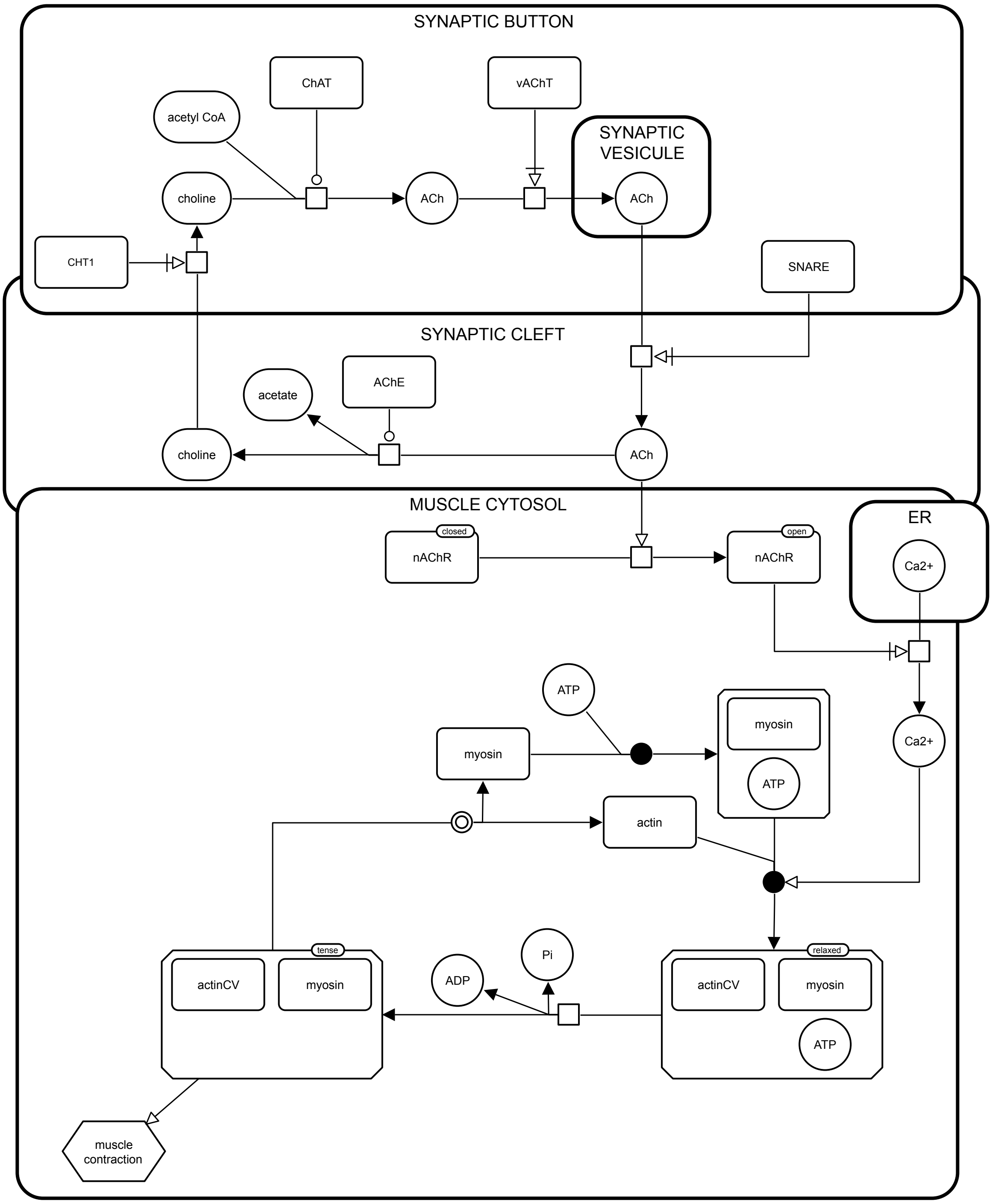

**Figure A.4:** *Neuronal/Muscle signalling. A description of inter-cellular signalling using SBGN.*

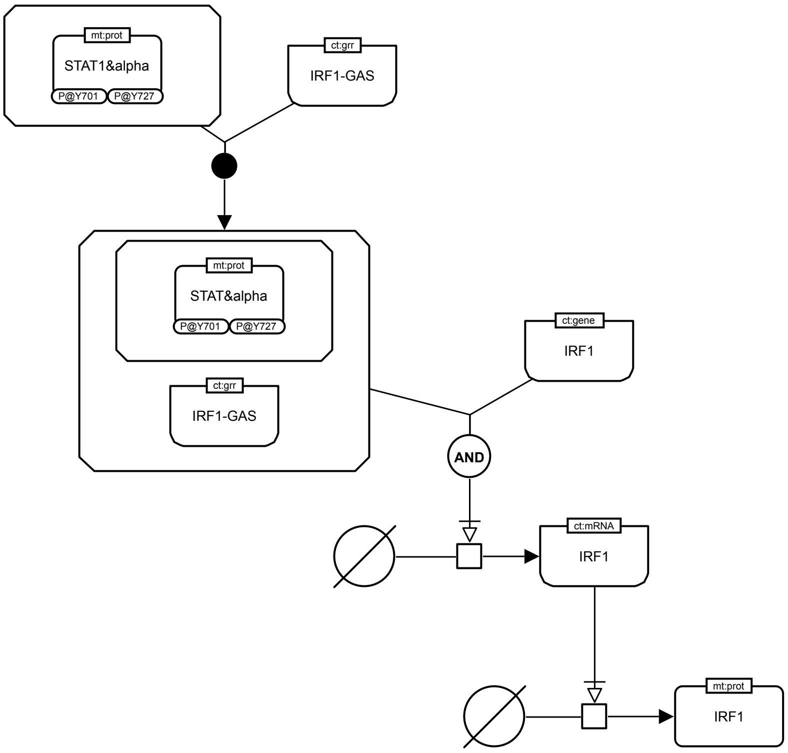

**Figure A.5:** *Activated STAT1$\alpha$ induction of the IRF1 gene. An example of gene regulation using logical operators.*

## Appendix B

## Examples of use of the *equivalence operator*

In the following, we give examples of use and misuse of the newly introduced *equivalence operator* (Section 2.9.4).

Figures B.1 and B.2 introduce the use of the *equivalence operator* to form generic *EPNs*. They show how the use of such an operator might allow reducing drastically the complexity of a map, without losing any information.

Figures B.3, B.4, and B.5 show misuses of the *equivalence operator*. The use of the *equivalence operator* requires respecting a number of rules (Section 3.5.6), that we recall briefly here: 1. Pools should not overlap; 2. A *process* should not affect *EPNs* belonging or deriving from both sides of an *equivalence operator*; 3. For *simple chemicals* (stateless *EPNs*) it is not allowed to show specifics both as input and as output of a pathway. The example of Figure B.3 on page 71 does not respect the first rule, the example of Figure B.5 on page 73 does not respect the first two rules, and the example of Figure B.5 on page 73 does not respect the third rule.

A

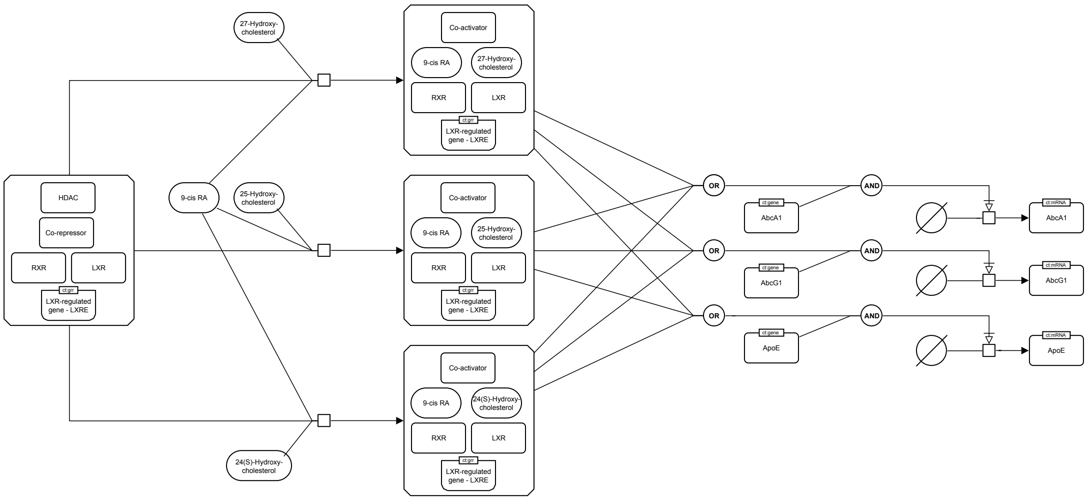

B

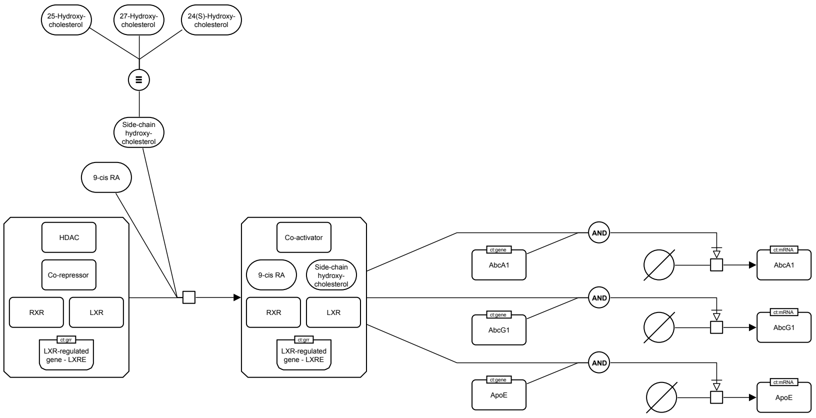

**Figure B.1:** *LXR signalling induced by 25-hydroxycholesterol, 27-hydroxycholesterol and 24(S)-hydroxycholesterol. A. Without an* equivalence operator*: events have to be multiplied for the three hydroxysterols. B. With an* equivalence operator*: a compact representation is possible allowing a better readability.*

A

B

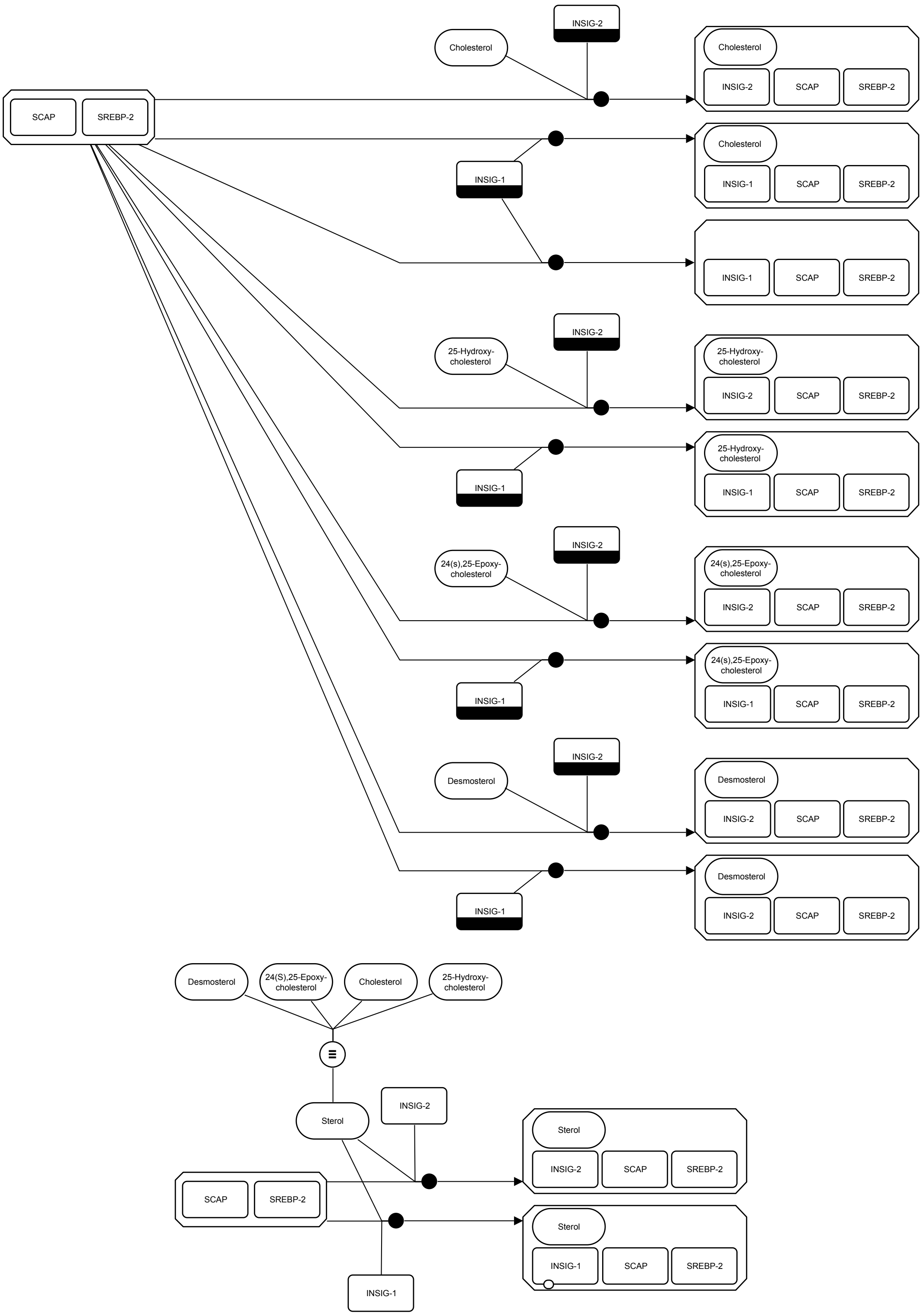

**Figure B.2:** *First step of the inhibition of cholesterol synthesis in presence of sterols. A. Without an* equivalence operator*. B. With an* equivalence operator*.*

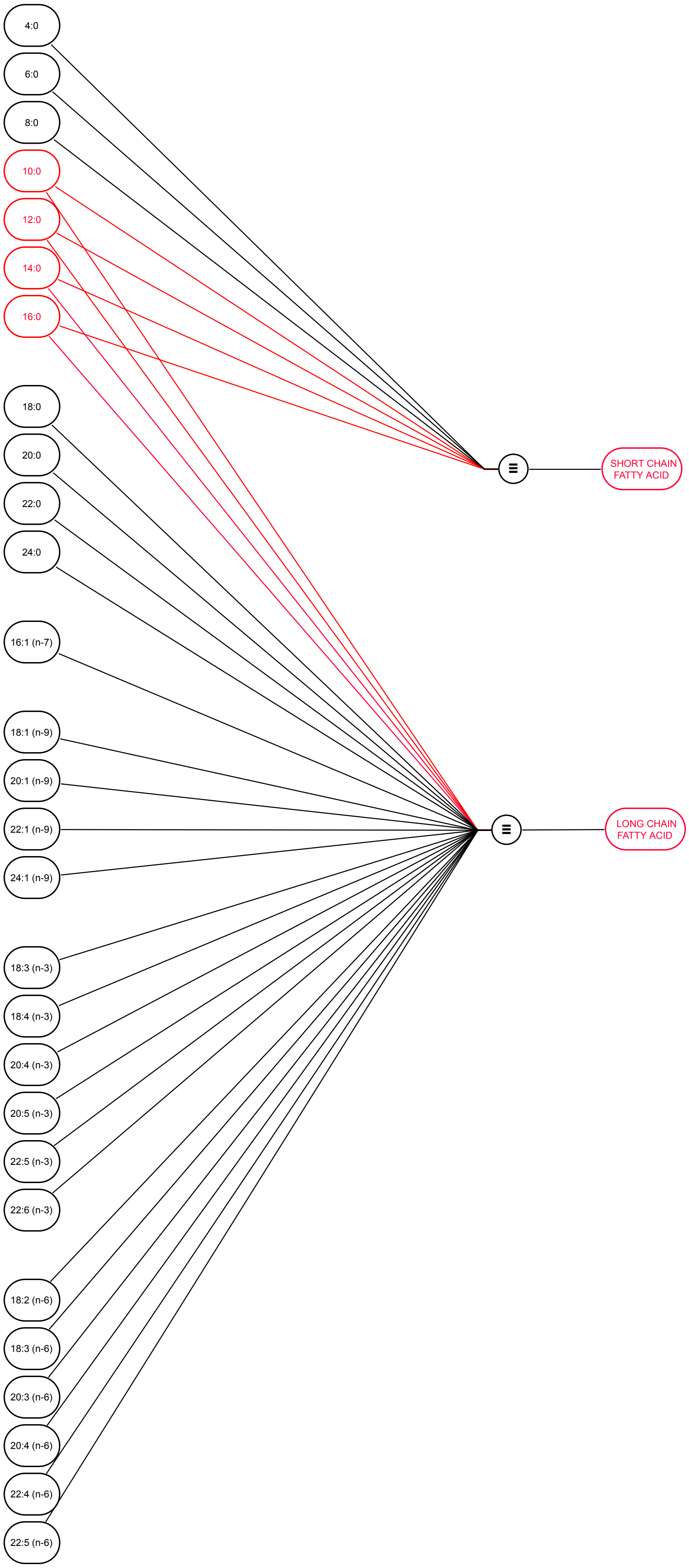

**Figure B.3:** *An example of misuse of the* equivalence operator. *The use of the* equivalence operators *does not respect the first rule, because the two generic pools colored in red on the right of the* equivalence operator *do overlap.*

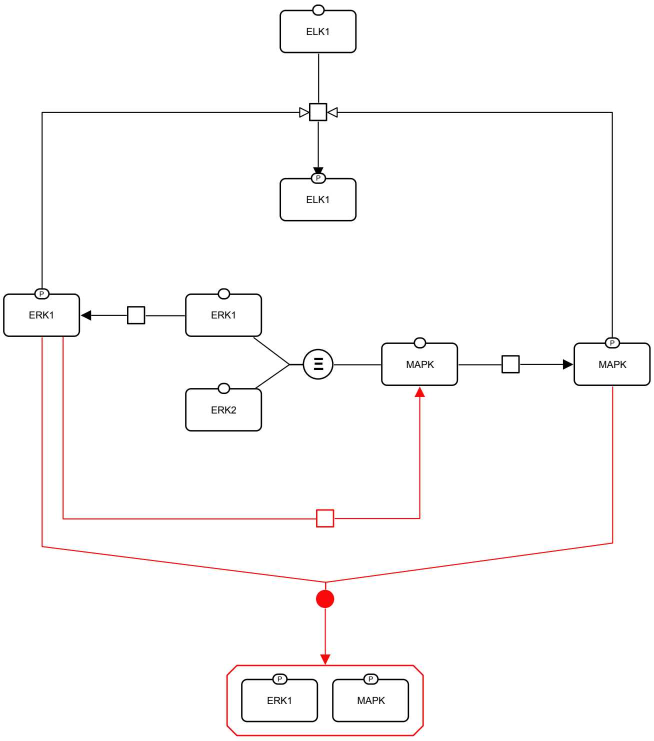

**Figure B.4:** *An example of misuse of the* equivalence operator *in a signalling pathway. Because the phosphorylation of ERK1 is 'included" in the phosphorylation of MAPK, phosphorylated ERK1 and phosphorylated MAPK are overlapping pools, which contradicts the first rule. As for the two processes marked in red, they both contradict the second rule, because phosphorylated ERK1 is on one side of the* equivalence operator*, while MAPK and phosphorylated MAPK are on its other side. In terms of automatic verification, phosphorylation of MAPK can be split into two processes: phosphorylation of ERK1 and phosphorylation of ERK2 since MAPK is equivalent to ERK1 and ERK2; then there would be a repeating process of ERK1 phosphorylation.*

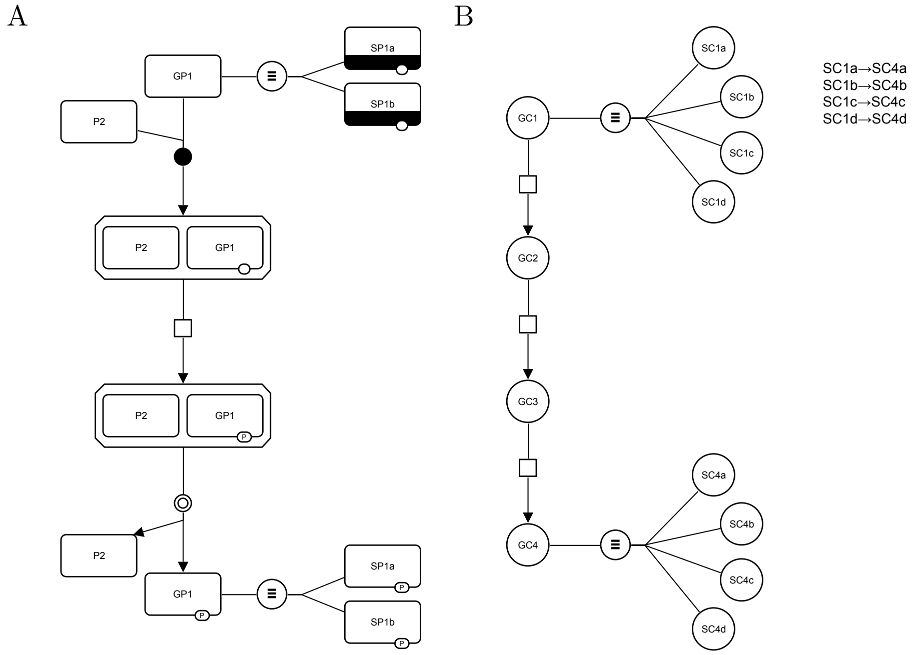

**Figure B.5:** *An example of misuse of the* equivalence operator *in an abstract pathway involving* simple chemicals*. A. Correct use. The use of the* equivalence operator *respects the third rule, because the pathway involves* macromolecules*, that are stateful* EPNs*. B. Misuse. The use of the* equivalence operator *does not respect the third rule, because the pathway involves* simple chemicals*, that are stateless* EPNs*.*

## Appendix C

## Reference card

Print the summary of SBGN symbols on the next page for a quick reference.

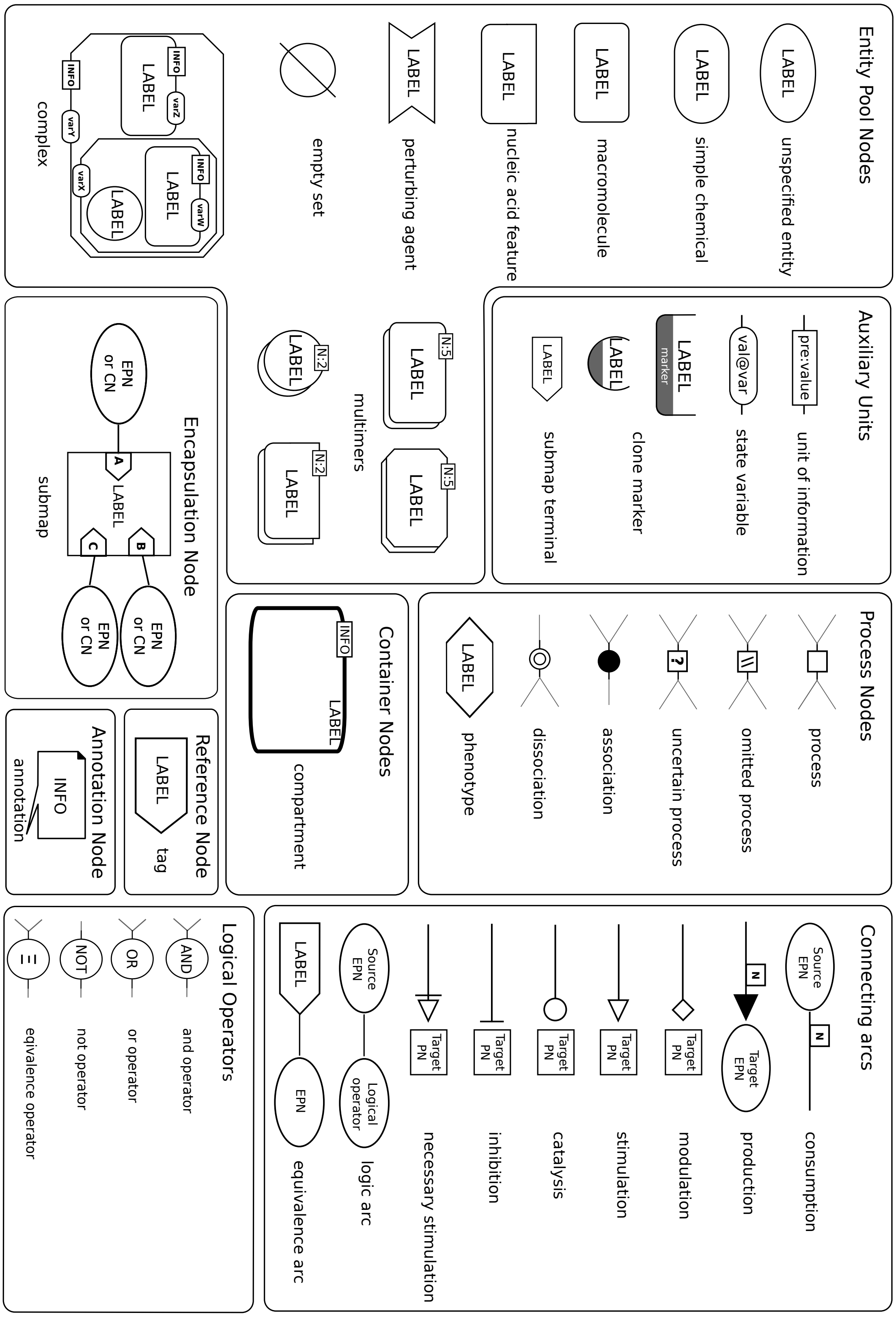

Entity Pool Nodes
LABEL
unspecified entity
LABEL
simple chemical
LABEL
macromolecule
LABEL
nucleic acid feature
LABEL
perturbing agent
empty set
INFO
LABEL
varZ
INFO
varY
INFO
LABEL
varW
varX
LABEL
complex
Auxiliary Units
pre:value
unit of information
val@var
state variable
LABEL
marker
LABEL
clone marker
LABEL
submap terminal
N:5
LABEL
N:5
LABEL
N:2
LABEL
N:2
LABEL
multimers
Encapsulation Node
EPN or CN
A
LABEL
B
C
EPN or CN
EPN or CN
submap
Process Nodes
process
omitted process
?
uncertain process
association
dissociation
LABEL
phenotype
Container Nodes
INFO
LABEL
compartment
Reference Node
LABEL
tag
Annotation Node
INFO
annotation
Connecting arcs
Source EPN
N
consumption
N
Target EPN
production
Target PN
modulation
Target PN
stimulation
Target PN
catalysis
Target PN
inhibition
Target PN
necessary stimulation
Source EPN
Logical operator
logic arc
LABEL
EPN
equivalence arc
Logical Operators
AND
and operator
OR
or operator
NOT
not operator
≡
eqivalence operator

## Appendix D

## Issues postponed to future levels

### D.1 Multicompartment entities

The problem of entities, such as macromolecules, spanning several compartments proved to be a challenge for the community involved in the development of SBGN Process Description Level 1. It was thus decided to leave it for a future Level. It turns out there is at the moment no obvious solution satisfactory for everyone. Three broad classes of solutions have been identified so far:

- One can systematically locate an *EPN* in a given *compartment*, for instance a transmembrane receptor in a membrane. However, the reactions of this entity with entities represented by *EPN* in other compartments, such as extracellular ligands and second messenger systems, will create artificial transport reactions.
- One can represent the domains of proteins in different compartments by *macromolecules*, and link all those macromolecules in a *complex* spanning several compartments. However, such a representation would be very confusing, implying that the domains are actually different molecules linked through non-covalent bonds.
- On can accept *macromolecules* that span several compartments, and represent domains as *units of information.* Those *units of information* should then be located in given compartments. To make a full use of such a representation, one should then start and end connecting arcs on given *units of information*, something prohibited by the current specification.

### D.2 Logical combination of state variable values

The value of a *state variable* has to be perfectly defined in SBGN Process Description Level 1. If a state variable can take the alternative values "A", "B" and "C", one cannot attribute it values such as "non-A", "A or B", "any" or "none". As a consequence some biochemical processes cannot be easily represented because of the very large number of states to enumerate. The decision to forbid such a Boolean logic lies in the necessity of maintaining truth path all over an SBGN map.

### D.3 Non-chemical entity nodes

The current specification cannot represent combinations of events and entities. For instance, a variable "voltage" cannot be controlled by a difference of concentration between different entities, such as a given ion in both sides of a membrane.

### D.4 State and transformation of compartments

In SBGN Process Description Level 1 a *compartment* is a stateless entity. It cannot carry *state variables*, and cannot be subjected to process modifying a state. As a result, a *compartment* cannot be transformed, moved, split or merged with another. If one wants to represent the transformation of a compartment, one has to create the start and end compartments, and represent the transport of all the *EPNs* from one to the other. This is not satisfactory, and should be addressed in the future.

## Appendix E

## Revision History

### E.1 Version 1.0 to Version 1.1

Below are the changes incorporated into Version 1.1 of the SBGN Process Description Level 1 specification.

| Description | Tracker ID |
|---|---|
| Regarding modulation of reversible processes, changed "should" to "must" be represented by two *process* | |
| Removed "The connectors and the box move as a rigid entity" in the definition of *process* | |
| Changed the definition of process node to "represent processes that transform one or several EPNs into *one or several EPNs, identical or different*" | |
| Changed SBO term of *compartment* From SBO:0000289 (functional compartment) to SBO:0000290 (physical compartment) | |
| Reoganised classification of glyphs | |
| Reoganised glyph section to reflect the above changes | |
| Revised reference card to reflect changes in glyph organisation | |
| Revised logic operators throughout spec to make sure input and output arcs meet before attaching to the glyph - as with processes. | |
| Added enumerated rules to grammar section. This is probably not complete, but should help the implementation of semantc validation by software tools. The hope is this will be refined as tools start validating maps. | |
| Updated UML maps and data dictionary to be consistent with rest of changes to spec. | |
| Definition of cardinality is ambiguous | 2840996 |
| *Sink and source* are lumped together | 2726435 |
| SBO terms are incorrect or missing. | 2841261 |
| *Compartment* description is confusing and contradictory. | 2841122 |
| *Clone marker* fill percentages unhelpful. | 2841114 |
| Use of CV for physical charactetistic not clear. | 2841085 |
| Definition of Cardinality is ambiguous. | 2840996 |
| input to AND on IFN example. | 2804326 |
| more SBO terms for *multimers* | 2803593 |
| Legend of figure 2.20 is incorrect | 2803537 |
| *continued on next page* | |

| *continued from previous page* | |
|---|---|
| Description | Tracker ID |
| legend of figure 3.2 | 2802990 |
| Compartment colouring | 2745703 |
| Errors in diag a4. | 2664912 |
| Change name of trigger glyph. | 2664908 |
| Transition should be renamed process. | 2664862 |
| Converting arcs tautological. | 2664843 |
| Example invalid. | 2545870 |
| consumption and production. | 2388317 |
| Should require circles to be distinguishable from ellipses | 2219388 |
| Figure 2.53 | 2162619 |
| Reference card: production | 2104471 |
| Figure 2.42 is wrong | 2104465 |
| Mistake in the multi-cellular example | 2395488 |
| Should not prevent processes having identical in and out | 2664933 |
| No description of linking to subunit rules. | 2545810 |
| Extensively revised the grammar section. The UML diagrams have been simplified to show glyph taxonomy, and the data dictionary has been pruned to just show glyph identity. The some syntax rules have been moved into semantics and the rules reformulated to make them easier to understand. | |
| Eliminated duplicate rules in layout section and revised text slightly. | |
| Phenotype cloning? | 2989007 |
| Perturbing agent description | 2940021 |

### E.2 Version 1.1 to Version 1.2

Below are the changes incorporated into Version 1.2 of the SBGN Process Description Level 1 specification.

| Description | Tracker ID |
|---|---|
| Perturbing agent description | 2940021 |
| Members of complex touching | 2849273 |
| PD Reference card error for submap glyph | 3029242 |

### E.3 Version 1.2 to Version 1.3

Below are the changes incorporated into Version 1.3 of the SBGN Process Description Level 1 specification.

| Description | Tracker ID |
|---|---|
| Incorrect editor on title page | |
| Typos in acknowledgements | |
| Fixed typo in item on catalysis in section 3.5.2.4. | |
| State variables figure 2.6 V1.2 | 3090543 |

### E.4 Version 1.3 to Version 2.0

Below are the changes incorporated into Version 2.0 of the SBGN Process Description Level 1 specification.

| Description | Tracker ID |
|---|---|
| Added equivalence operator glyph (Section 2.9.4) | |
| Added annotation glyph (Section 2.11.1) | |
| Added submap terminal glyph (Section 2.3.5) | |
| Replaced source and sink glyphs by empty set glyph (Section 2.4.7) | |
| Added subunit glyphs (Section 2.3.4) | |
| Changed state variable glyph from ellipse to stadium (Section 2.3.2) | |
| Changed simple chemical glyph from circle to stadium (Section 2.4.2) | |
| Set process glyph identity to “instance” (Section 3.2) | |
| Broke arcs section into three new sections: flux arcs (Section 2.7), modulation arcs (Section 2.8) and logic arc (Section 2.10) | |
| Moved sections “Referring to other nodes and arcs” (Section 2.12) and “Encapsulation” (Section 2.13) to the end of chapter 2 | |
| Homogenized “Container”, “Label” and “Auxiliary units” entries for all glyphs | |
| Redrew all figures with SBGN-ED using a unified style | |
| Added “Incoming arcs” and “Outgoing arcs” entries for all nodes | |
| Updated the reference card with the new glyphs and shapes (appendix C) | |
| Added acknowledgements (Section 5) | |
| Rewrote the standfirst and the “Label” entry of the state variable section (Section 2.3.2) | |
| Added text to the standfirst of the multimer section and table 2.8 showing the different glyphs (Section 2.4.5) | |
| Rewrote standfirst of the tag section (Section 2.12.1) | |
| Rewrote standfirst of the submap section, some text explaining the examples and captions of the figures (Section 2.13.1) | |
| Moved equivalence arc section (Section 2.12.2) to section “Encapsulation” (Section 2.13) and rewrote the standfirst | |
| Added a standfirst to the logical operators section (Section 2.9) | |
| Rewrote the definitions of the and operator (Section 2.9.1), the or operator (Section 2.9.2) and the not operator (Section 2.9.3) glyphs | |
| Updated figure 3.1, table 3.1, table 3.2, and the tables of section 3.4.1 and 3.4.2 with the new glyphs | |
| Added rules regarding the equivalence operator glyph in the semantic rules section (Section 3.5.6) | |
| Added usage examples for the equivalence operator glyph in a new appendix (appendix B) | |
